# Health inequalities for China’s low-income population: trends, subgroup differences, and influencing factors, 2010–2022

**DOI:** 10.3389/fpubh.2025.1569726

**Published:** 2025-04-10

**Authors:** Qiwei Feng, Ying Wang, Xinbin Xia

**Affiliations:** School of Humanities and Management, Hunan University of Chinese Medicine, Changsha, China

**Keywords:** health inequality, low-income population, recentered influence function, RIF-Oaxaca decomposition, China

## Abstract

**Objective:**

Health inequality is a global challenge, with low-income populations often facing higher health risks. This study aims to systematically analyze the current status, trends, and influencing factors of health inequalities for China’s low-income population.

**Methods:**

Utilizing panel data from the China Family Panel Studies (CFPS) from 2010 to 2022, the low-income population was identified using a threshold of 67% of median income. Health inequalities were measured across four dimensions: self-rated health, mental health, two-week health, and chronic diseases status, using the Erreygers Index (EI) and Wagstaff Index (WI). Recentered Influence Function (RIF) regression and RIF-Oaxaca decomposition were employed to examine influencing factors of health inequalities and sources of disparities across urban–rural, gender, and age dimensions.

**Results:**

From 2010 to 2022, the degree of health inequality was significantly higher for the low-income group compared to the middle and high-income groups in China. Inequalities in self-rated health and chronic diseases status showed an increasing trend for the low-income population. Per capita household income (PCHI) was a key factor, exhibiting a significant negative impact on inequalities in self-rated health and mental health (*p* < 0.01). Age had an inverted U-shaped effect on health inequalities, while household size significantly and negatively influenced disparities in self-rated health and two-week health (*p* < 0.01). Differences in the level of medical expertise of the visited institutions significantly affected chronic disease status inequalities (*p* < 0.01). The PCHI was the primary source of health inequality disparities across urban–rural, gender, and age groups, with the older adult low-income group experiencing significantly higher levels of health inequality compared to the non-older adult group.

**Conclusion:**

Health inequalities for the low-income population in China are becoming increasingly severe, particularly pronounced among older adult and rural groups. The study recommends implementing interventions across multiple dimensions, including income support, healthcare accessibility, and family care support, while adopting differentiated policies tailored to the characteristics of various groups. Particular attention should be given to intersectionally disadvantaged groups such as low-income older adult individuals in rural areas.

## 1 Introduction

Health is the foundation of individual well-being and social development. Health inequalities not only affect personal welfare but also relate to social cohesion and economic growth, posing a significant global challenge. Health inequalities refer to the systematic, avoidable, and unjust differences in health outcomes that exist between different populations, between different social groups within the same population, or among populations ranked by social status (1). This phenomenon is widespread both within and between countries (2–4). Despite the remarkable progress made by countries in recent years in improving population health, inequalities in health outcomes resulting from disparities in health resource allocation and socioeconomic factors persist and are widening (5). Particularly in countries with rapid economic development, the expansion of income gaps often accompanies the exacerbation of health inequalities (6). The United Nations Sustainable Development Goals advocate for achieving universal health coverage, emphasizing that everyone should have access to quality essential healthcare services and essential medicines, and promoting equality within and among countries (4). In response to this global initiative, countries have successively implemented multiple healthcare reforms and policies to promote health equity.

As the world’s largest developing country, China’s experience in addressing health inequalities has important implications for other emerging economies. In 2016, China proposed the “Healthy China 2030” initiative, aiming to comprehensively improve the health of the entire population and significantly enhance health equity (7). To achieve this goal, China has established a social basic medical insurance system covering the entire population, with the medical insurance participation rate reaching 95% by the end of 2023 (8). Simultaneously, China has promoted the reform of the tiered diagnosis and treatment system (classifying diseases according to severity and urgency and the difficulty of treatment, with healthcare institutions at different levels responsible for treating different diseases) and the reform of the medical insurance payment model to promote the equalization of basic healthcare services (9, 10).

However, despite these efforts driving the overall improvement of the health status of Chinese residents, health inequalities remain prominent, with the low-income group experiencing significantly worse health outcomes compared to middle and high-income groups. According to data from the Sixth National Health Service Survey conducted by the National Health Commission of the People’s Republic of China, in 2018, the two-week prevalence of illness among the low-income group (41.1%) was significantly higher than that of the high-income group (28.7%). Moreover, the non-poor low-income group had the lowest average hospitalization expense reimbursement ratio, at only 52.5% (compared to 58.5% for the high-income group), and the proportion of household medical expenditure to income for the low-income group was more than three times that of the high-income group (11). The Low-income group not only significantly lag behind the high-income group in self-rated health, chronic disease prevalence, and life expectancy but this gap is also widening (12). In 2018, the disparities in two-week prevalence of illness and chronic disease prevalence between the low-income and high-income groups in China increased by 10.3 and 11.2 percentage points, respectively, compared to 1993 (11).

Existing studies have explored the issue of health inequalities from various perspectives. Firstly, a substantial body of research has focused on the relationship between income and health, revealing a significant association between income inequality and health outcomes. Studies have shown that even after controlling for other socioeconomic factors, the risk of developing health problems among the low-income group is three times higher than that of the high-income group (13). A study in Colombia found that the association between income inequality and poor health status was more pronounced among the low-income group (6). Research in China has revealed that the low-income population experiences suboptimal health outcomes and face financial barriers to accessing healthcare services, perpetuating a vicious cycle of poor health and economic disadvantage (14). Longitudinal studies in the United States have also found that as income inequality worsens, life expectancy among low-income groups stagnates or even declines in some populations (5). Furthermore, income inequality not only affects inequalities in physical health but also significantly impacts inequalities in mental health, with economic status contributing up to 44.7% of the socioeconomic inequalities in mental health (15).

Research has identified that, in addition to income, health inequalities are influenced by numerous factors. A cross-national study by Li et al. (16) found that individual marital status, education level, and healthcare infrastructure were significantly associated with health inequalities. A study in rural western China showed that income and education were the main sources of health inequalities (17). Researchers have extensively explored the impact of social capital on health inequalities, finding that social capital can buffer the negative effects of poverty on health (18, 19). The role of healthcare insurance systems has also received considerable attention. Studies have found that the impact of healthcare insurance systems on health inequalities is rather complex. The implementation of instant medical reimbursement policies has significantly increased the hospitalization utilization rate among migrant populations, promoting health equity (20). However, in rural China, the urban and rural resident basic medical insurance may exacerbate health inequalities between different income groups (21). Some studies have also found that health insurance has no significant effect on health inequalities among the older adult (22). Spatial environmental differences are also important factors influencing health inequalities (23, 24). This is because the quality of the environment, such as access to healthcare services, water, and sanitation facilities, is consistently better in urban areas and eastern regions of China compared to rural areas and central and western regions (23). Factors such as living conditions, accessibility of healthcare services, household size, and household composition can all affect health inequalities (22, 24).

When measuring health inequalities, the Concentration Index (CI) and its variants, such as the Wagstaff Index (WI) and Erreygers Index (EI), are the most commonly used methods to assess the unequal distribution of health outcomes across socioeconomic status (17, 20–22, 24, 25). Some studies have also employed the Gini coefficient (26), Kakwani index (19), relative index of inequality, and slope index of inequality (23) to measure health inequalities. In analyzing the influencing factors, existing studies primarily adopt the following approaches: Firstly, health variables are used as dependent variables to construct regression models, such as logistic regression and ordered logistic regression, to identify key factors influencing health inequalities (19, 27). Secondly, health inequality indices are used as dependent variables, employing Wagstaff decomposition to quantify the contribution of different factors to health inequalities (17, 21, 22) or utilizing Oaxaca-Blinder decomposition to analyze the sources of health disparities between groups (28). A few scholars have also employed Recentered Influence Function (RIF) regression to examine the distributional characteristics of health inequalities (24).

Despite the multidimensional and multi-perspective exploration of health inequalities in the existing literature, several shortcomings remain. Firstly, current research often focuses on health inequalities between countries, urban and rural areas, regions, and specific populations (such as the older adult, pregnant women, and children), while systematic attention to the low-income population is relatively insufficient, particularly lacking long-term trend analyses based on panel data. Secondly, existing studies tend to concentrate on a single health dimension, lacking a comprehensive examination of multidimensional health outcomes such as self-rated health, mental health, two-week health, and chronic diseases status. Thirdly, in terms of methodological application, most studies have employed traditional methods such as the CI and its decomposition, as well as Shapley decomposition, while few have adopted newer methods like the RIF regression and RIF-Oaxaca decomposition, which have advantages over traditional methods, including fewer assumptions and restrictions, and easier estimation and interpretation. Fourthly, a systematic analysis of the multiple factors influencing health inequalities for the low-income population, such as socioeconomic status characteristics, demographic characteristics, psychological attitudes, and health behaviors, requires further in-depth investigation.

As China’s economy has grown rapidly, health inequalities between different income groups have become increasingly prominent. Clarifying the current status, trends, and influencing factors of health inequalities for the low-income population in China is not only a theoretical issue that urgently needs to be addressed by academia but also a practical challenge that the government must tackle. Based on this, we utilize data from the China Family Panel Studies (CFPS) from 2010 to 2022, employing a threshold of 67% of median income to define the low-income population, and applying methods such as the EI and WI to systematically examine the current status and evolution of health inequalities for the low-income population in China across four dimensions: self-rated health, mental health, two-week health, and chronic diseases status. Furthermore, this study will comprehensively employ econometric methods such as the RIF regression and RIF-Oaxaca decomposition to conduct an in-depth analysis of the factors influencing health inequalities for the low-income population from multiple dimensions, including socioeconomic status characteristics, demographic characteristics, psychological attitudes, health behaviors, and structural factors. The research findings will not only contribute to revealing the current state and influencing factors of health inequalities for the low-income population in China, providing empirical evidence for formulating targeted health intervention policies, but also offer valuable insights into understanding the challenges of health equity during the transitional period of emerging economies, thereby contributing to the promotion of global health equity and social justice.

## 2 Materials and methods

### 2.1 Data sources and preprocessing

This study utilized data from the China Family Panel Studies (CFPS), a nationally representative, longitudinal survey conducted by the Institute of Social Science Survey at Peking University. The CFPS is carried out every two years, covering 25 provinces, municipalities, and autonomous regions, representing more than 95% of the Chinese population. The study utilizes a complex sampling design, involving multiple stages and levels, with probability proportional to size. Appropriate weights are assigned to each observation. To ensure the representativeness of our study findings, we applied standardized sampling weights to CFPS data across all years. Since standardized weights were not available for the period between 2010 and 2016, we performed weight standardization after data processing. Additionally, we matched data across different years and CFPS sub-databases using family and individual identifiers to construct the analytical dataset. Regarding missing data and sample attrition, we followed the CFPS official manual to determine the causes and nature of missing values. For variables that remain stable over time (e.g., gender), we replaced missing values with observed data from adjacent years. For randomly missing variables (e.g., self-rated health), we prioritized imputations using highly correlated variables or logically consistent values from adjacent years. If, after imputation, a variable still exhibited a high proportion of missing values (above 10%), it was excluded from the analysis. If the missing rate was minimal (below 0.1%), the corresponding cases were directly removed to maintain the completeness and representativeness of the dataset. Additionally, as with most longitudinal studies, panel sample attrition was present in our dataset due to individual migration, mortality, refusal, or loss to follow-up. Given that we strictly applied the CFPS-provided individual panel standardized weights, we believe that the impact of sample attrition on the robustness of our analysis is limited. Ultimately, we constructed two main types of databases. The first type consisted of seven cross-sectional databases from 2010 to 2022. We first calculated the annual median per capita household income using weighted data from the family economic database. From the individual-level database, we then constructed two samples: one representing the national population and another for the low-income population. The national sample was used to compare the differences in health inequalities between the low-income group and other groups, while the low-income population sample was used to analyze the current status and trends of internal health inequalities. These cross-sectional data were weighted using the individual database’s cross-sectional standardized weights for the corresponding year. The sample sizes of the low-income population from 2010 to 2022 were 10,587, 9,685, 10,086, 11,157, 9,261, 6,631, and 5,981, respectively, totaling 63,389 individuals. The national sample sizes were 31,173, 29,444, 29,158, 29,703, 25,803, 19,967, and 18,176, respectively, totaling 183,423 individuals. The second type was an unbalanced panel data of the low-income population from 2016 to 2022 (with sample sizes of 10,747, 7,646, 5,466, and 4,911, respectively), weighted using individual panel standardized weights, and employed to construct RIF regression and decomposition models to explore the factors influencing health inequalities for the low-income population.

### 2.2 Variables

#### 2.2.1 Core dependent variables

The core dependent variables were health inequality indices. Considering data availability and existing research, we selected four health variables from multiple perspectives, including subjective and objective, long-term and short-term, and mental and physical health: self-rated health (24, 29) (represents respondents’ overall perception of their health status), mental health (27), two-week health (whether physical discomfort occurred within two weeks) (11), and chronic disease status (13, 17, 30) (whether suffering from chronic diseases in the past six months). We measured health inequality indices using the PCHI as the ranking variable (31). It should be noted that due to changes in the CFPS questionnaire design, the self-rated health and mental health variables in some years were only suitable for horizontal comparison and not for longitudinal comparison. The classification of self-rated health in 2010 differs slightly from that in 2012–2022, making it incomparable for direct longitudinal analysis with other years. Regarding the measurement of mental health, CFPS employed two different scales across survey years: the K6 scale in 2010 and 2014, and the CESD-8 scale in 2012 and 2016–2022. Since these two scales have different scoring criteria and cannot be converted between each other, we processed the data from different periods separately. For the CESD-8 scale (2012, 2016–2022), following the research of Briggs et al. (32), we reassigned the options of the eight items to scores of 0–3, and a total score of ≥9 was defined as mentally unhealthy and assigned a value of 0. For the K6 scale (2010 and 2014), referring to Furukawa et al. (33), the items were reassigned to scores of 4–0 and summed. Based on the results of Sakurai et al. (34), a score of ≥5 was defined as mentally unhealthy and assigned a value of 0. In our trend analysis, we analyzed these two periods separately, while for panel data analysis, we only used data from 2016–2022 (where the same scale was consistently used) to ensure measurement consistency.

#### 2.2.2 Explanatory variables

According to the conceptual framework of social determinants of health proposed by the World Health Organization (WHO) (35), health equity is influenced by structural determinants and intermediary determinants. Structural determinants encompass socioeconomic and political context (governance, policies, cultural values, etc.) and socioeconomic position (education, occupation, income, gender, ethnicity, etc.), which affect health equity through intermediary determinants (material circumstances, behaviors and biological factors, psychosocial factors, and the health system). Although the WHO framework emphasizes the pathways between these factors, this study primarily focuses on examining the overall impact of various factors on health inequalities rather than verifying specific causal mechanisms. Based on data availability, model stability considerations, and relevant literature, we simplified and adapted the WHO framework through rigorous statistical tests (such as chi-square tests and correlation coefficient analysis) to select variables with the strongest explanatory power and lowest multicollinearity as parallel explanatory variables in our analysis. Specifically, socio-demographic characteristics included age (36), gender (37), ethnicity, marital status (14, 38), and household size (22, 28, 38), reflecting an individual’s basic social attributes, which are usually beyond personal control. Considering the potential nonlinear relationship between age and the dependent variable, and to avoid multicollinearity, we applied mean-centering (the difference between age and the sample mean of 48.54) and quadratic terms of age in the panel data models. Socioeconomic status characteristics included urban–rural attribute, years of education (37), agricultural occupation (agricultural or non-agricultural) (39), personal income (PI), and per capita household income (PCHI) (40), which can be changed to a certain extent by individual efforts and reflect an individual’s position in the socioeconomic hierarchy. Material circumstances (41, 42) included the source of cooking water (Cooking water source) and the type of cooking fuel (Cooking fuel type, i.e., whether the cooking fuel was clean energy such as bottled gas, liquefied gas, natural gas, piped gas, solar energy, biogas, or electricity). Health behavior variables (43) included whether smoking in the past month (Past-month smoking) (28, 44), whether drinking alcohol more than three times per week in the past month (Frequent past-month alcohol use) (45), whether having a nap habit, and whether exercising weekly (14, 46). Psychological and attitude variables included life satisfaction and self-rated social status (26, 35). Structural factors included medical insurance reimbursement ratio (MIRR), basic social medical insurance enrollment (21, 22), and the visited institution’s level of medical expertise (patients’ evaluation of their visited healthcare institutions, excluding medical service, where medical service encompasses conditions of doctors, medicine, hospitalizations, travel distance and transportation convenience) (14). The specific variables and their assignments are shown in Table 1.

**Table 1:** Variable assignment and characteristics of the low-income population in China, 2016–2022.

Variable	Assignment	2016		2018		2020		2022	
		N/mean	%/SE	N/mean	%/SE	N/mean	%/SE	N/mean	%/SE
Self-rated health status									
Unhealthy	1	2,055	19.12	1,685	22.04	1,007	18.43	906	18.44
Average	2	2,025	18.84	1,127	14.74	705	12.89	494	10.06
Relatively healthy	3	3,219	29.95	2,649	34.64	1,958	35.82	1,917	39.03
Very healthy	4	2,018	18.78	1,169	15.29	937	17.14	807	16.43
Extremely healthy	5	1,430	13.31	1,016	13.29	859	15.72	788	16.04
Mental health status									
Unhealthy	0	2,525	23.5	2,082	27.22	1,536	28.11	1,384	28.18
Healthy	1	8,222	76.5	5,564	72.78	3,930	71.89	3,527	71.82
2-week health status									
Healthy	0	7,317	68.08	4,898	64.06	3,857	70.56	3,455	70.36
Unhealthy	1	3,430	31.92	2,748	35.94	1,609	29.44	1,456	29.64
Chronic disease status									
Without chronic diseases	0	8,922	83.02	6,337	82.87	4,597	84.11	4,076	83.01
With chronic disease	1	1,825	16.98	1,309	17.13	869	15.89	834	16.99
Residence									
Rural	0	6,267	58.31	4,526	59.19	3,219	58.89	2,821	57.44
Urban	1	4,480	41.69	3,120	40.81	2,247	41.11	2,090	42.56
Gender									
Female	0	5,286	49.19	4,042	52.87	2,824	51.66	2,512	51.16
Male	1	5,461	50.81	3,604	47.13	2,642	48.34	2,398	48.84
Ethnicity									
Han Chinese	1	8,809	81.96	6,559	85.78	4,629	84.68	4,264	86.84
Other	2	1,939	18.04	1,087	14.22	837	15.32	646	13.16
Marital status									
Single	1	1,638	15.24	1,013	13.26	861	15.76	843	17.17
Married/Cohabiting	2	8,111	75.47	5,810	75.98	4,132	75.6	3,626	73.85
Divorced/Widowed	3	998	9.29	823	10.76	472	8.64	441	8.98
Agricultural occupation									
Non-agricultural	0	5,620	52.29	3,815	49.89	2,888	52.83	2,738	55.75
Agricultural	1	5,127	47.71	3,831	50.11	2,579	47.17	2,173	44.25
Past-month smoking									
No	0	7,689	71.54	5,525	72.26	4,007	73.31	3,674	74.82
Yes	1	3,058	28.46	2,121	27.74	1,459	26.69	1,237	25.18
Frequent past-month alcohol use									
No	0	9,065	84.35	6,421	83.98	4,734	86.61	4,269	86.93
Yes	1	1,682	15.65	1,225	16.02	732	13.39	642	13.07
Nap habit									
No	0	5,641	52.49	3,346	43.77	2,162	39.56	1,899	38.67
Yes	1	5,106	47.51	4,300	56.23	3,304	60.44	3,012	61.33
Weekly exercise									
No	0	7,106	66.12	4,458	58.31	4,232	77.42	3,466	70.58
Yes	1	3,641	33.88	3,188	41.69	1,234	22.58	1,445	29.42
Life satisfaction									
dissatisfied/Neutral	1	4,999	46.51	2,362	30.89	1,661	30.39	1,538	31.33
Somewhat satisfied	2	2,954	27.48	2,149	28.1	1,699	31.08	1,582	32.21
Very satisfied	3	2,795	26	3,135	41.01	2,107	38.54	1,790	36.46
Self-rated social status									
Relatively low	1	3,761	35	1,935	25.3	1,322	24.19	1,175	23.94
Average	2	4,587	42.68	3,159	41.32	2,383	43.6	2,151	43.8
Relatively high	3	2,400	22.33	2,552	33.37	1,761	32.21	1,585	32.27
Cooking water source									
Non-tap water	0	3,706	34.48	2,481	32.45	1,436	26.26	1,202	24.48
Tap water	1	7,041	65.52	5,165	67.55	4,031	73.74	3,709	75.52
Cooking fuel type									
Non-clean fuels	0	4,962	46.17	2,915	38.13	1,686	30.84	1,260	25.66
Clean fuels	1	5,785	53.83	4,731	61.87	3,780	69.16	3,650	74.34
Basic social medical insurance enrollment									
Not enrolled	0	928	8.63	680	8.9	583	10.66	470	9.56
Enrolled	1	9,820	91.37	6,966	91.1	4,883	89.34	4,441	90.44
Level of medical expertise of the visited institution									
Low	1	793	7.38	969	12.67	553	10.11	600	12.22
Fair	2	5,910	54.99	2,555	33.41	1,586	29.01	1,166	23.74
High	3	4,044	37.63	4,122	53.91	3,328	60.87	3,145	64.04
PI (CNY)		6483.7	259.09	8053.97	253.73	9064.55	300.17	12248.7	393.03
PCHI (CNY)		5875.86	114.6	7670.36	120.63	8407.97	127.66	10149.3	155.64
MIRR		0.1	0	0.11	0.01	0.1	0.01	0.1	0.01
Household size (Persons)		4.62	0.14	4.43	0.11	4.48	0.12	4.19	0.09
Years of education		6.12	0.19	6.2	0.17	6.89	0.18	7.33	0.14
Age (years)		47.43	0.57	49.65	0.58	48.84	0.66	48.91	0.62
Total		10747.2		7646.01		5466.28		4910.78	

SE, Standard Error; PI, Personal Income; PCHI, Per Capita Household Income; MIRR, Medical Insurance Reimbursement Ratio; CNY, Chinese yuan.

### 2.3 Definition of low-income population

We used the per capita annual household net income from the CFPS family economic database as the PCHI. After weighting for population size and income, we calculated the median PCHI for each year. We adopted a relative standard, using 67 and 200% of the median PCHI as thresholds to divide the population into three categories: low-income group (below 67% of the median), middle-income group (between 67 and 200%), and high-income group (above 200%). The threshold of 67% of the median income for defining the low-income group is primarily based on mainstream international practices in relative poverty research, such as the OECD standard, which commonly uses 50 to 75% of the median income as the poverty line (47), as well as relevant domestic studies in China (48, 49). This threshold effectively identifies vulnerable groups at actual risk of health inequality, avoiding the potential drawbacks of overly narrow classification with a 50% threshold or an excessively broad categorization with a 75% threshold. To facilitate subsequent analyses, we generated a binary income group variable to distinguish whether a sample belongs to the low-income group. For analyses focusing on the low-income population as a whole, we refer to it as the low-income population. When comparing income groups or discussing subgroups within the low-income category, we use the term ‘group’.

### 2.4 Measurement of health inequalities: Erreygers index, and Wagstaff index

Considering that the health variables used in this study are ordinal categorical variables, we selected the Erreygers Index (EI) (50) and the Wagstaff Index (WI) (51) to measure inequalities in four health dimensions from 2010 to 2022 (52). Both indices are improvements on the traditional Concentration Index (CI) (53), with EI being an absolute inequality index adjusted for bounded variables and WI being a relative inequality index adjusted for bounded variables (54). We chose EI as the primary measurement indicator and used WI for robustness tests, mainly based on the following considerations. First, EI satisfies scale invariance and mirror consistency, making the degree of inequality between different health indicators comparable (55). Second, as an absolute inequality index, EI is not affected by changes in the average level of the health variable, making it more suitable for comparisons across time periods and groups. Finally, when dealing with binary or ordinal categorical health variables, EI can avoid the influence of the bounded variable effect (56). Although WI does not satisfy the mirror condition, it provides a complementary verification from the perspective of relative inequality (57). The consistency of the results from the two indices can increase the reliability of the research conclusions, while differences suggest that health inequalities may have multidimensional characteristics.

The specific calculation formulas (50) are shown in Equations 1, 2.


(1) $$EI=\frac{8}{N^{2}\left(b-a\right)}\sum_{i=1}^{N}z_{i}h_{i}$$

(2) $$WI=\frac{2\left(b-a\right)}{N^{2}\left(b-\mu_{h}\right)\left(\mu_{h}-a\right)}\sum_{i=1}^{N}z_{i}h_{i}$$

In these equations, EI is the Erreygers Index, and WI is the Wagstaff Index. N is the sample size, and $h_{i}$ is the health level of the i -th individual. $\sum_{i=1}^{N}z_{i}h_{i}$ expresses the rank-dependence character, which is a weighted sum of all individual health levels, where $z_{i}=\frac{n+1}{2}-r_{i}$ , and $r_{i}$ is the relative rank of the i -th individual after sorting by per capita annual household net income (rank divided by sample size N ). $\mu_{h}$ is the mean of the health variable h , while b and a are the upper and lower bounds of the health variable h , respectively. For both indices, a positive value indicates that the health distribution favors groups with higher socioeconomic status, while a negative value indicates the opposite. A value of zero represents a completely equal health distribution. The larger the absolute value of the index, the higher the degree of health inequality.

### 2.5 RIF regression for health inequalities

To explore the influencing factors of health inequalities among the low-income population, we conducted RIF regression analysis based on four waves of panel data from 2016 to 2022. RIF is the re-centered influence function method developed by Firpo et al. (58). Heckley et al. (54) introduced it into the field of health inequality research, and Rios Avila (59) further improved the RIF regression and its decomposition method. Compared with traditional concentration index decomposition methods, RIF regression has many advantages. It directly decomposes the weighted covariance of health and socioeconomic rank, more accurately explaining socioeconomic-related health inequalities. It is applicable to various inequality indices (such as EI, WI, etc.), expanding the scope of research. It has fewer assumptions and restrictions, effectively reducing endogeneity bias caused by omitted variables. Its results are easier to estimate and interpret. It can simultaneously examine the impact of multiple explanatory variables on health inequalities, providing a more comprehensive analytical perspective. RIF regression is performed in two steps:

Step 1: Calculate the RIF value of the health inequality indices


(3) $$RIF{\left(\mathrm{health},\mathrm{index}\right)}_{i}=\mathrm{index}+IF_{i}^{\mathrm{index}}$$

In this equation, index is the health inequality indices, which is EI or WI in this study, and the calculation methods are shown in Equations 1, 2. $IF_{i}^{\mathrm{index}}$ is the influence function of the index on individual i , capturing the impact of a small perturbation at the health level $h_{i}$ on the inequality index. The specific form of the influence function differs for different inequality indices, and the specific calculation methods and formulas can be found in Heckley et al. (54).

Step 2: Construct the RIF regression model


(4) $$RIF{\left(\mathrm{health},\mathrm{Index}\right)}_{\mathrm{it}}=\beta_{0}+\underset{j}{\sum }\beta_{j}x_{jit}+\gamma_{i}+u_{\mathrm{pt}}+\varepsilon_{\mathrm{it}}$$

$RIF{\left(\mathrm{health},\mathrm{index}\right)}_{\mathrm{it}}$ is the RIF value of the index for individual i at time t . $\mu_{h}$ and $h_{i}$ are explained in the same way as in Equation 1. $\beta_{0}$ is the constant term. $x_{jit}$ represents the value of the j -th explanatory variable for individual i at time t , which are the same as the other variables mentioned above, i.e., the independent variables that may affect health inequalities. $\beta_{j}$ is the coefficient of the j -th explanatory variable. If $\beta_{j}$ is positive, it indicates that an increase in $x_{jit}$ will widen the degree of health inequality, and vice versa. $\gamma_{i}$ is the individual fixed effect, capturing the unobservable factors at the individual level. $u_{\mathrm{pt}}$ represents the province-year fixed effect, capturing all unobservable factors that vary over time at the province level and all unobservable factors that vary across provinces over time. $\varepsilon_{\mathrm{it}}$ is the random error term for individual i . The specific derivation process can be found in Nie et al. (60) and Heckley et al. (54).

To ensure the reliability of the estimates, we performed difference tests (chi-square tests between categorical variables and analysis of variance between categorical and continuous variables) and multicollinearity tests on all independent variables during the model construction process. At the same time, we included multidimensional fixed effects to control for the influence of unobservable factors. The reasons for choosing province-level fixed effects rather than city or community fixed effects are twofold. First, policy formulation and implementation in China mainly occur at the provincial level, with policy differences primarily manifesting between provinces and between urban and rural areas. Second, introducing city fixed effects would not only significantly increase the complexity of the model but would also be limited by data availability.

### 2.6 RIF-Oaxaca decomposition for health inequalities

To analyze the sources of health inequality differences within the low-income population across dimensions such as urban–rural, gender, and age (with a cutoff at 60 years old), we combined RIF with Oaxaca-Blinder decomposition, following the research of Firpo et al. (61) and Rios-Avila (62). This combined approach not only retains the advantages of RIF regression but also enables accurate decomposition of the sources of health inequality differences between groups. To control for the influence of time trends, such as changes in the macroeconomic environment and policies, on health inequalities, we included year fixed effects in the decomposition model. The reason for not incorporating individual fixed effects is that introducing a large number of individual effects in RIF-Oaxaca decomposition would not only lead to complex model calculations and convergence difficulties but also absorb individual-level variability, affecting the identification of determinants of health inequalities. Simultaneously, considering that province fixed effects may exhibit collinearity with key covariates and that regional differences themselves are important sources of health inequalities, we did not include province fixed effects in this study. The analysis process is as follows:

Step 1: Construct group regression models.

For a binary group variable T (such as urban–rural, gender, age), let T = 1 represent the first group (e.g., rural, female, under 60 years old) and T = 2 represent the second group (e.g., urban, male, 60 years old and above). For each group, establish an RIF regression model that includes year fixed effects and calculate the expected value of the health inequality indices. The models for the first and second groups are shown in Equations 5, 6, respectively.


(5) $$v_{1}=E\left[RIF\left(h_{i},v\left(F_{H\backslash T=1}\right)\right)\right]=\beta^{1}{\bar{X}}^{1}+\delta^{1}D+\varepsilon^{1}$$

(6) $$v_{2}=E\left[RIF\left(h_{i},v\left(F_{H\backslash T=2}\right)\right)\right]=\beta^{2}{\bar{X}}^{2}+\delta^{2}D+\varepsilon^{2}$$

$v_{1}$ and $v_{2}$ represent the inequality index 𝑣 (such as EI or WI) for the first and second groups, respectively. $E\left[\cdot \right]$ denotes the expected value. $h_{i}$ represents the health level of the i -th individual. $v\left(F_{H\backslash T=1}\right)$ is the value of the health inequality index 𝑣 for the first group, and $v\left(F_{H\backslash T=2}\right)$ is the value of the health inequality index 𝑣 for the second group. $\beta^{1}$ and $\beta^{2}$ are the regression coefficient vectors for the two groups, respectively. ${\bar{X}}^{1}$ and ${\bar{X}}^{2}$ are the mean vectors of the covariates (independent variables) for the two groups, representing sample characteristics. $\delta^{1}$ and $\delta^{2}$ are the year fixed effects coefficient vectors for the corresponding groups. D is the matrix of year dummy variables (shared by all groups). $\varepsilon^{1}$ and $\varepsilon^{2}$ are the error terms for the corresponding groups.

Step 2: Construct the counterfactual group.

Construct the counterfactual inequality index $v_{c}$ , assuming that the first group has the regression coefficients of the second group:


(7) $$v_{c}=\beta^{2}{\bar{X}}^{1}+\delta^{2}D$$

Step 3: Decompose the total difference, i.e., the difference between the inequality indices of the two groups, and decompose it into the coefficient effect and the composition effect.


(8) $$\begin{array}{l} \Delta =v_{1}-v_{2}=\underset{\Delta v_{s}}{\underset{︸}{v_{1}-v_{c}}}+\underset{\Delta v_{x}}{\underset{︸}{v_{c}-v_{2}}}=\underset{\Delta v_{s}}{\underset{︸}{\left(\beta^{1}-\beta^{2}\right){\bar{X}}^{1}+\left(\delta^{1}-\delta^{2}\right)D}}+\\ \underset{\Delta v_{x}}{\underset{︸}{\beta^{2}\left({\bar{X}}^{1}-{\bar{X}}^{2}\right)}} \end{array}$$

Δ is the total effect of the group difference, which can be decomposed into $\Delta v_{s}$ and $\Delta v_{x}\text{.}$ $\Delta v_{s}$ is the coefficient effect, reflecting the “treatment difference” in health inequalities between the two groups when characteristics such as education and occupation are the same, capturing some unobservable factors and representing the unexplained portion. $\Delta v_{x}$ is the composition effect, reflecting the contribution of differences in characteristics such as education and occupation between the two groups to health inequalities, representing the explained portion.

Step 4: Decompose the contributions of each covariate.


(9) $$\Delta v_{x,j}=\beta_{j}^{2}\left({\bar{X}}_{j}^{1}-{\bar{X}}_{j}^{2}\right)$$

(10) $$\Delta v_{s,j}=\left(\beta_{j}^{1}-\beta_{j}^{2}\right){\bar{X}}_{j}^{1}$$

$\beta_{j}^{1}$ and $\beta_{j}^{2}$ are the coefficients of the j -th covariate in the regressions for the first and second groups, respectively. ${\bar{X}}_{j}^{1}$ and ${\bar{X}}_{j}^{2}$ are the means of the j -th covariate in the first and second groups, respectively.

### 2.7 Analytical strategy

All data in this study were processed and analyzed using Stata 17.0. The significance test level was set at 0.05.

## 3 Results

### 3.1 Basic characteristics of China’s low-income population

**Figure 1:**
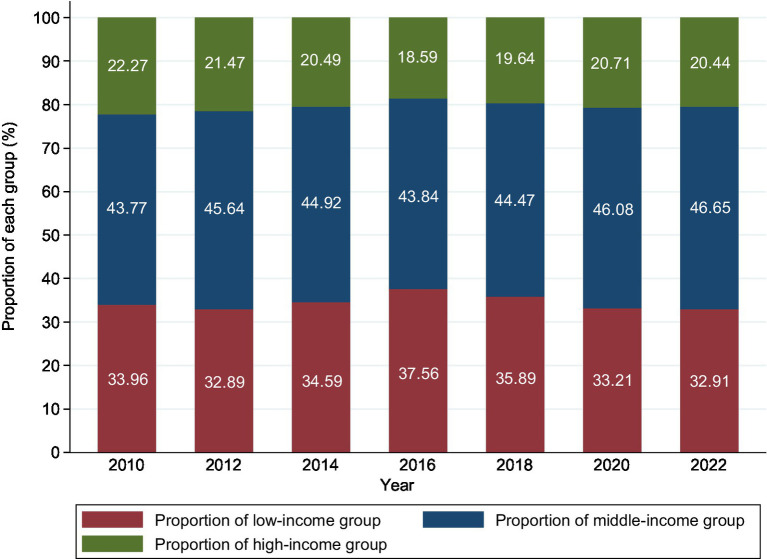
Proportion of each income group in China from 2010 to 2022.

**Figure 2:**
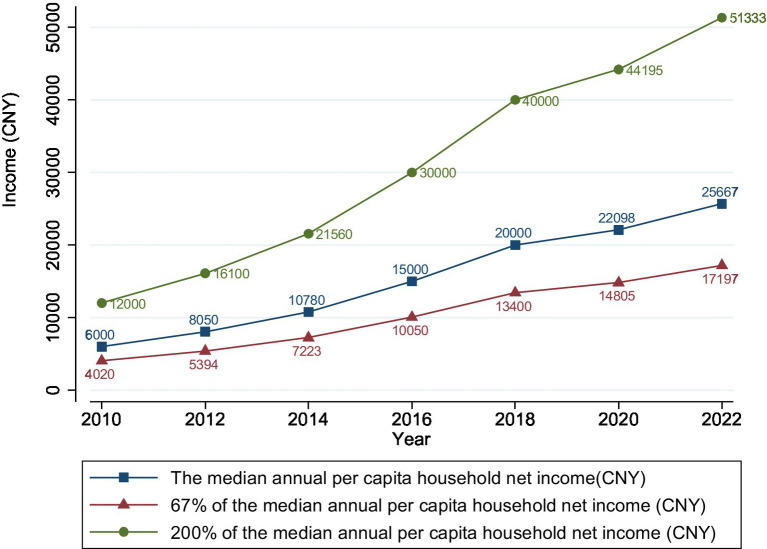
The annual per capita household net income in China from 2010 to 2022.

From 2010 to 2022, the proportion of China’s low-income population in the total population followed an inverted U-shaped trend, reaching a peak of 37.56% in 2016 and then gradually declining to 32.91% in 2022 (Figure 1). Although the PCHI of Chinese residents showed an upward trend, the dividing line for the middle-and high-income groups (200% of the median PCHI) rose faster than the upper limit of income for the low-income group (67% of the median PCHI), indicating a widening gap between the rich and the poor (Figure 2). Additionally, the growth rate of the PCHI slowed down from 2018 to 2022, with a slight improvement during 2020–2022, though not reaching the pre-2018 growth rates.

The changes in health status for the low-income population varied across different dimensions (Table 2). Self-rated health showed an improving trend, with the proportion of positive evaluations (relatively healthy, very healthy, extremely healthy) rising from 60.85% in 2012 to 71.23% in 2022. However, mental health continuously deteriorated, with the proportion of healthy individuals in 2022 decreasing by 7.09 percentage points compared to 2012. The proportion of healthy individuals showed a U-shaped pattern in terms of two-week health status with 2018 as the turning point, while the proportion of individuals without chronic diseases showed a slight overall decline. The trends in various health dimensions for the low-income group were generally consistent with the national situation, but the health levels remained lower than the national average. For example, in 2022, the proportion of positive self-rated health evaluations for the low-income group was 6.08 percentage points lower than the national level (Table S1).

**Table 2:** Health status and trends of low-income population in China, 2010–2022.

Variable	2010		2012		2014		2016		2018		2020		2022	
	N/mean	%/SE	N/mean	%/SE	N/mean	%/SE	N/mean	%/SE	N/mean	%/SE	N/mean	%/SE	N/mean	%/SE
Self-rated health status														
Unhealthy	281	2.66	1,951	20.14	1,892	18.76	2,313	20.73	1,967	21.24	1,171	17.66	1,110	18.56
Average	1,158	10.93	1,840	19	1,515	15.02	2,156	19.32	1,294	13.97	824	12.43	611	10.21
Relatively healthy	676	6.39	2,914	30.09	3,086	30.59	3,334	29.88	3,334	36	2,415	36.41	2,356	39.38
Very healthy	3,591	33.92	2,065	21.32	2,156	21.38	1,972	17.67	1,388	14.98	1,114	16.8	956	15.98
Extremely healthy	4,881	46.1	915	9.44	1,437	14.25	1,382	12.39	1,279	13.81	1,107	16.69	949	15.86
Mental health status														
Unhealthy	511	4.82	2,075	21.42	500	4.96	2,676	23.98	2,461	26.58	1,842	27.77	1,705	28.51
Healthy	10,077	95.18	7,610	78.58	9,586	95.04	8,481	76.02	6,800	73.42	4,790	72.23	4,276	71.49
2-week health status														
Healthy	7,798	73.65	6,812	70.33	6,886	68.27	7,365	66.01	5,955	64.31	4,726	71.28	4,184	69.95
Unhealthy	2,790	26.35	2,873	29.67	3,201	31.73	3,792	33.99	3,305	35.69	1,905	28.72	1,797	30.05
Chronic disease status														
Without chronic diseases	9,183	86.73	8,607	88.86	8,542	84.69	9,067	81.27	7,743	83.61	5,631	84.91	4,931	82.45
With chronic disease	1,405	13.27	1,079	11.14	1,545	15.31	2,089	18.73	1,518	16.39	1,001	15.09	1,050	17.55
Total	10,587		9,685		10,086		11,157		9,261		6,631		5,981	

SE, standard error.

Regarding socioeconomic characteristics (Table 1), the PI and PCHI of the low-income population increased by 1.89 times and 1.73 times, respectively, between 2016 and 2022. The average years of education increased by 1.21 years, and the proportion of agricultural workers decreased by 3.46 percentage points. In terms of psychological attitudes, the proportion of individuals with low self-rated social status and low life satisfaction (dissatisfied/neutral) decreased by 11.06 and 15.19 percentage points, respectively, in 2022 compared to 2016. Regarding health behaviors and material circumstances, the proportion of individuals with a nap habit and those using tap water and clean fuel increased, while smoking and drinking behaviors decreased. In terms of sociodemographic characteristics, there was a trend towards smaller household sizes. Regarding structural factors, approximately 90% of the population was enrolled in basic social medical insurance, but the participation rate slightly decreased. Regarding the level of medical expertise of the visited institution, the evaluations showed a polarization, with the proportion of evaluations as high and low increasing by 26.41 and 4.85 percentage points, respectively. The MIRR remained at around 11%.

### 3.2 Status and trends of health inequalities for the low-income population in China

Given the high consistency in direction, significance, and trend changes between EI and WI, we primarily focused on analyzing EI results.

From 2010 to 2022, except for the chronic disease dimension from 2010 to 2014, significant pro-rich health inequalities existed in China (*p* < 0.05). Between 2016 and 2022, apart from mental health, significant differences in health inequalities between the low-income and middle-to-high-income groups were generally observed in the other three dimensions (*p* < 0.05). For example, in the self-rated health dimension in 2022, the between-group Z-value of EI was −3.05 (*p* < 0.0001). The extent of health inequalities among the low-income group was significantly higher than that of the middle-to-high-income group. In 2022, the EI for self-rated health in the low-income group was 0.07 (*p* < 0.0001), significantly higher than the middle-to-high-income group (0.014, *p* > 0.05) and the national level (0.051, *p* < 0.0001). From 2012 to 2022, although the inequality indices for self-rated health and chronic disease status of the low-income group fluctuated, they showed an overall upward trend, reflecting a deepening of health inequalities (Table 3).

**Table 3:** National health inequality indices in China, 2010–2022.

Health variable	Year	National		Low-income		Middle-High-income	
		WI	EI	WI	EI	WI	EI
Self-rated health status	2022	0.051****	0.051****	0.070****	0.070****	0.014	0.014
		−0.011	−0.011	−0.016	−0.016	−0.009	−0.009
	z-value	−3.040***	−3.050***				
	2020	0.045***	0.045***	0.059****	0.059****	0.013	0.013
		−0.012	−0.012	−0.013	−0.013	−0.01	−0.01
	z-value	−2.780**	−2.800**				
	2018	0.059****	0.059****	0.072****	0.071****	0.003	0.003
		−0.01	−0.01	−0.016	−0.016	−0.01	−0.01
	z-value	−3.690***	−3.690***				
	2016	0.040***	0.040***	0.065***	0.064***	0.003	0.003
		−0.012	−0.012	−0.016	−0.016	−0.008	−0.008
	z-value	−3.360***	−3.350***				
	2014	0.058****	0.057****	0.033*	0.033*	0.023**	0.023**
		−0.011	−0.011	−0.014	−0.014	−0.008	−0.008
	z-value	−0.6	−0.61				
	2012	0.051***	0.051***	0.035	0.035	0.033****	0.033****
		−0.013	−0.013	−0.024	−0.024	−0.008	−0.008
	z-value	−0.1	−0.09				
	2010	0.123****	0.075****	0.082****	0.057****	0.058****	0.032****
		−0.016	−0.009	−0.017	−0.012	−0.012	−0.007
	z-value	−1.15	−1.86				
Mental health status	2022	0.138****	0.100****	0.093***	0.076***	0.087***	0.059***
		−0.017	−0.012	−0.027	−0.022	−0.021	−0.014
	z-value	−0.16	−0.66				
	2020	0.159****	0.107****	0.077**	0.062**	0.048*	0.028*
		−0.024	−0.016	−0.025	−0.02	−0.022	−0.013
	z-value	−0.87	−1.41				
	2018	0.150****	0.099****	0.117****	0.091****	0.021	0.012
		−0.021	−0.014	−0.018	−0.014	−0.021	−0.012
	z-value	−3.390***	−4.160****				
	2016	0.206****	0.123****	0.146****	0.107****	0.107****	0.054****
		−0.02	−0.012	−0.028	−0.021	−0.019	−0.009
	z-value	−1.15	−2.320*				
	2014	0.200****	0.028****	0.072	0.014	0.155***	0.017***
		−0.037	−0.005	−0.046	−0.009	−0.038	−0.004
	z-value	1.38	0.39				
	2012	0.178****	0.098****	0.058**	0.039**	0.124****	0.059****
		−0.017	−0.009	−0.019	−0.013	−0.02	−0.009
	z-value	2.380*	1.25				
	2010	0.245****	0.030****	0.115*	0.021*	0.129**	0.012**
		−0.033	−0.004	−0.051	−0.009	−0.04	−0.004
	z-value	0.22	−0.94				
2-week health status	2022	0.087****	0.068****	0.099****	0.083****	0.022	0.016
		−0.015	−0.011	−0.021	−0.017	−0.016	−0.012
	z-value	−2.930**	−3.180**				
	2020	0.083****	0.062****	0.065**	0.053**	0.008	0.006
		−0.016	−0.012	−0.022	−0.018	−0.019	−0.013
	z-value	−2.000*	−2.160*				
	2018	0.114****	0.097****	0.107****	0.098****	0.033	0.026
		−0.016	−0.014	−0.018	−0.016	−0.018	−0.015
	z-value	−2.900**	−3.270**				
	2016	0.078***	0.066***	0.069**	0.062**	0.003	0.003
		−0.02	−0.017	−0.025	−0.022	−0.017	−0.014
	z-value	−2.190*	−2.270*				
	2014	0.058***	0.048***	−0.012	−0.01	0.026	0.02
		−0.016	−0.013	−0.02	−0.017	−0.016	−0.013
	z-value	1.48	1.44				
	2012	0.03	0.024	0.008	0.007	0.023	0.019
		−0.018	−0.014	−0.028	−0.023	−0.015	−0.012
	z-value	0.46	0.44				
	2010	0.067***	0.049***	0.061**	0.047**	0.032*	0.023*
		−0.017	−0.013	−0.023	−0.018	−0.015	−0.01
	z-value	−1.04	−1.19				
Chronic disease status	2022	0.055**	0.028**	0.096**	0.055**	−0.021	−0.01
		−0.02	−0.011	−0.033	−0.019	−0.023	−0.011
	z-value	−2.890**	−2.950**				
	2020	0.063**	0.029**	0.081*	0.042*	0.015	0.007
		−0.023	−0.011	−0.032	−0.016	−0.026	−0.011
	z-value	−1.61	−1.75				
	2018	0.034	0.017	0.069**	0.038**	−0.04	−0.019
		−0.019	−0.01	−0.024	−0.013	−0.021	−0.01
	z-value	−3.340***	−3.360***				
	2016	<0.001	<0.001	0.059*	0.036*	−0.03	−0.018
		−0.023	−0.014	−0.03	−0.018	−0.02	−0.012
	z-value	−2.490*	−2.490*				
	2014	−0.007	−0.004	−0.01	−0.005	−0.031	−0.016
		−0.023	−0.012	−0.023	−0.012	−0.024	−0.012
	z-value	−0.64	−0.63				
	2012	−0.004	−0.002	−0.012	−0.005	−0.019	−0.007
		−0.019	−0.007	−0.027	−0.011	−0.019	−0.007
	z-value	−0.23	−0.22				
	2010	0.028	0.012	0.051	0.023	−0.025	−0.01
		−0.022	−0.009	−0.029	−0.013	−0.019	−0.008
	z-value	−2.210*	−2.190*				

*p < 0.05, **p < 0.01, ***p < 0.001, ****p < 0.0001.

Table S2 revealed the differences in health inequalities within the low-income population in terms of urban–rural, gender, and age dimensions. The differences in health inequalities between urban and rural groups, as well as between genders, were not statistically significant. However, age-related disparities were significant in certain dimensions and years. Overall, except for chronic disease status, the EI values for all subgroups across other health dimensions have remained positive and generally significant since 2016. This indicates that while the between-group differences were relatively small, within-group health inequalities were more pronounced. Specifically, in the urban–rural dimension, from 2012 to 2022, the self-rated health inequality indices of the rural low-income group showed an upward trend (EI increased from 0.018 to 0.076) and consistently exceeded that of the urban group since 2014. From 2016 to 2020, the mental health inequality indices of both urban and rural low-income groups showed a downward trend, with the rural group experiencing a higher degree of inequality than the urban group. However, in 2022, the inequality level of the urban low-income group began to surpass that of the rural group. From a gender perspective, between 2012 and 2022, the self-rated health inequality levels of both males and females generally showed an upward trend. From 2016 to 2020, the mental health inequality levels of both genders declined year by year but rebounded in 2022. Starting from 2020, the two-week health inequality level of males exceeded that of females. Since 2016, the chronic disease status inequality indices of both genders increased annually, with males being higher than females, but females began to surpass males in 2022. In the age dimension, the health inequality level of the older adult low-income group (aged 60 and above) was generally higher than that of the non-older adult group, with this difference being most significant in the mental health dimension. In 2022, the EI for mental health was 0.114 (*p* < 0.001) for the older adult group and 0.047 (*p* > 0.05) for the non-older adult group. From 2018 to 2022, the mental health inequality level of the older adult group continued to rise, and their two-week health inequality level was also significantly higher than that of the non-older adult group. Furthermore, from 2020 onwards, while the older adult group maintained strong statistical significance in mental health inequality, other groups showed decreased significance levels. Similarly, urban areas experienced a notable decrease in significance levels for both self-rated health and mental health inequality from 2020, with 2020 showing no statistical significance. During 2018–2020, the significance levels of two-week health inequality across all groups changed, though the direction of the indices remained unchanged, with these changes becoming less pronounced in 2022.

### 3.3 Factors influencing health inequalities for the low-income population in China

Table 4 showed that the absolute values of WI regression coefficients were slightly higher than those of EI, but the direction, significance, and relative magnitude among variables remained consistent. This indicated that regardless of whether EI or WI was used, the identified factors influencing health inequality were robust and reliable. To simplify the analysis, the following discussion only focused on the model results with EI as the dependent variable.

**Table 4:** RIF regression results of health inequalities for the low-income population in China, 2016–2022.

Variable	Self-rated health status		Mental health status		2-week health status		Chronic disease status	
	EI	WI	EI	WI	EI	WI	EI	WI
LnPI	0.002 (0.003)	0.002 (0.003)	−0.003 (0.004)	−0.004 (0.005)	0.006 (0.004)	0.007 (0.005)	0.001 (0.003)	0.001 (0.006)
LnPCHI	−0.048*** (0.018)	−0.049*** (0.018)	−0.085*** (0.022)	−0.109*** (0.027)	−0.035 (0.023)	−0.038 (0.026)	−0.027 (0.018)	−0.052* (0.031)
LnMIRR	<0.001 (0.001)	<0.001 (0.001)	−0.001 (0.002)	−0.001 (0.003)	<−0.001 (0.002)	−0.001 (0.003)	0.002 (0.002)	0.001 (0.003)
Age	0.031 (0.028)	0.030 (0.028)	0.046 (0.039)	0.062 (0.052)	0.021 (0.046)	0.021 (0.052)	−0.008 (0.021)	−0.018 (0.038)
Age^2^	−0.001*** (<0.001)	−0.001*** (<0.001)	<−0.001 (<0.001)	<−0.001 (<0.001)	−0.001*** (<0.001)	−0.001*** (<0.001)	−0.001*** (<0.001)	−0.001*** (<0.001)
Residence (reference group: rural)								
Urban	−0.081 (0.059)	−0.078 (0.059)	0.079 (0.071)	0.104 (0.093)	0.030 (0.079)	0.035 (0.090)	0.138** (0.055)	0.250*** (0.097)
Gender (reference group: female)								
Male	0.364** (0.172)	0.364** (0.174)	0.876*** (0.262)	1.105*** (0.334)	0.510* (0.307)	0.587* (0.346)	−0.118 (0.300)	−0.252 (0.494)
Ethnicity (reference group: other ethnicities)								
Han Chinese	0.118 (0.131)	0.131 (0.133)	0.190 (0.179)	0.229 (0.235)	0.382 (0.350)	0.438 (0.423)	0.047 (0.134)	0.069 (0.246)
Marital status (reference group: married/cohabiting)								
Single	−0.217** (0.098)	−0.219** (0.099)	−0.268* (0.154)	−0.326 (0.198)	−0.216 (0.170)	−0.242 (0.191)	−0.124 (0.081)	−0.185 (0.142)
Divorced/Widowed	−0.009 (0.078)	−0.010 (0.079)	−0.122 (0.114)	−0.188 (0.146)	0.054 (0.114)	0.055 (0.129)	0.104 (0.083)	0.186 (0.146)
Years of education	0.009 (0.008)	0.009 (0.008)	0.016* (0.010)	0.021* (0.013)	0.012 (0.012)	0.014 (0.013)	<0.001 (0.008)	<0.001 (0.014)
Agricultural occupation (reference group: non-agricultural)								
Agricultural	−0.024 (0.030)	−0.025 (0.030)	0.015 (0.048)	0.020 (0.062)	−0.025 (0.048)	−0.024 (0.054)	−0.069* (0.041)	−0.124* (0.072)
Self-rated social status (reference group: average)								
Relatively low	0.008 (0.021)	0.008 (0.022)	0.025 (0.031)	0.021 (0.040)	−0.009 (0.032)	−0.008 (0.037)	0.028 (0.025)	0.049 (0.044)
Relatively high	−0.026 (0.021)	−0.025 (0.021)	−0.023 (0.031)	−0.036 (0.040)	−0.002 (0.034)	<−0.001 (0.038)	−0.050** (0.025)	−0.089** (0.044)
Past-month smoking (reference group: no)								
Yes	0.050 (0.040)	0.048 (0.039)	0.064 (0.054)	0.090 (0.069)	0.077 (0.055)	0.086 (0.063)	−0.003 (0.047)	−0.003 (0.085)
Frequent past-month alcohol use (reference group: no)								
Yes	−0.014 (0.031)	−0.017 (0.031)	−0.006 (0.045)	−0.005 (0.059)	−0.018 (0.048)	−0.020 (0.055)	−0.005 (0.037)	−0.008 (0.066)
Nap habit (reference group: no)								
Yes	−0.017 (0.020)	−0.015 (0.020)	0.035 (0.030)	0.044 (0.039)	0.028 (0.031)	0.033 (0.035)	0.010 (0.024)	0.013 (0.043)
Weekly exercise (reference group: no)								
Yes	−0.003 (0.021)	−0.003 (0.021)	0.030 (0.029)	0.034 (0.038)	−0.041 (0.032)	−0.049 (0.036)	0.003 (0.024)	−0.001 (0.043)
Life satisfaction (reference group: somewhat satisfied)								
Dissatisfied/Neutral	0.015 (0.020)	0.016 (0.020)	0.021 (0.032)	0.013 (0.041)	0.019 (0.032)	0.020 (0.037)	0.027 (0.026)	0.043 (0.046)
Very satisfied	0.024 (0.022)	0.025 (0.022)	0.014 (0.030)	0.022 (0.038)	−0.047 (0.032)	−0.051 (0.036)	−0.040 (0.025)	−0.078* (0.045)
Cooking water source (reference group: non-tap water)								
Tap water	−0.043 (0.026)	−0.042 (0.026)	−0.086** (0.038)	−0.107** (0.049)	−0.098** (0.039)	−0.111** (0.044)	−0.052 (0.034)	−0.092 (0.060)
Cooking fuel type (reference group: non-clean fuels)								
Clean fuels	0.017 (0.026)	0.018 (0.026)	−0.040 (0.036)	−0.051 (0.047)	0.060 (0.037)	0.070* (0.042)	−0.002 (0.031)	−0.004 (0.055)
Household size	−0.035*** (0.008)	−0.035*** (0.008)	0.008 (0.012)	0.010 (0.015)	−0.033*** (0.013)	−0.037** (0.014)	−0.012 (0.010)	−0.020 (0.017)
Basic social medical insurance enrollment (reference group: not enrolled)								
Enrolled	<−0.001 (0.033)	0.001 (0.033)	0.014 (0.049)	0.022 (0.063)	0.007 (0.050)	0.011 (0.057)	0.067** (0.034)	0.116* (0.061)
Level of medical expertise of the visited institution (reference group: fair)								
Low	0.029 (0.031)	0.027 (0.031)	0.070 (0.047)	0.085 (0.060)	0.017 (0.049)	0.011 (0.056)	0.118*** (0.039)	0.209*** (0.070)
High	0.007 (0.019)	0.006 (0.019)	0.015 (0.029)	0.019 (0.037)	−0.011 (0.029)	−0.012 (0.033)	0.020	0.031 (0.042)
							(0.023)	
Constant	0.588** (0.241)	0.577** (0.242)	0.068 (0.311)	0.097 (0.400)	0.128 (0.426)	0.110 (0.499)	0.539** (0.272)	1.023** (0.476)
Individual FE	Yes	Yes	Yes	Yes	Yes	Yes	Yes	Yes
Province FE	Yes	Yes	Yes	Yes	Yes	Yes	Yes	Yes
Year FE	Yes	Yes	Yes	Yes	Yes	Yes	Yes	Yes
RIF Mean	0.072	0.072	0.059	0.076	0.082	0.095	0.038	0.067
Within R^2^	0.018	0.018	0.008	0.008	0.011	0.010	0.012	0.012
N	23,262	23,262	23,262	23,262	23,262	23,262	23,262	23,262

*, **, and *** denote significance at the 10%, 5%, and 1% levels, respectively. PI, Personal Income; PCHI, Per Capita Household Income; MIRR, Medical Insurance Reimbursement Ratio.

**Figure 3:**
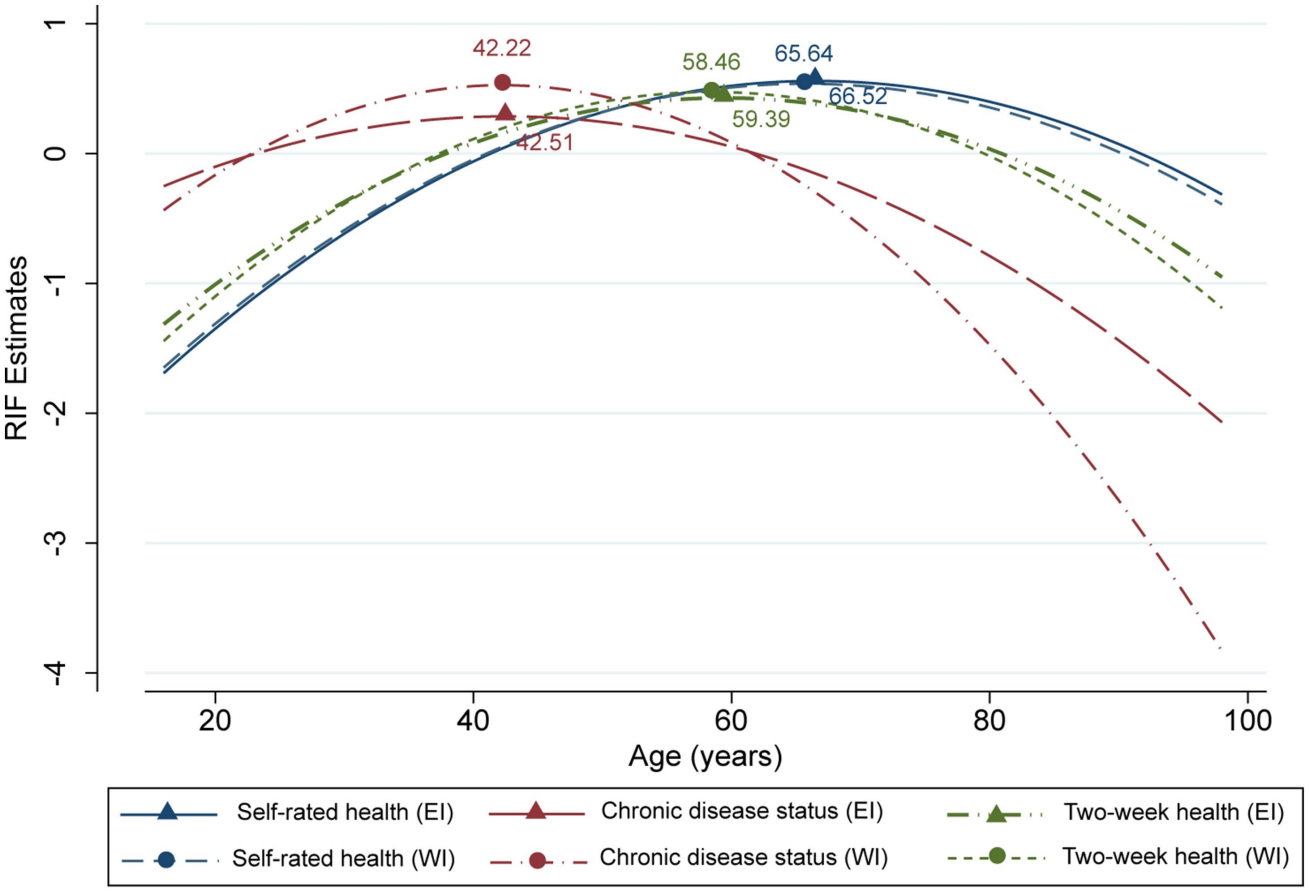
Age effects on different types of health inequalities.

In terms of sociodemographic characteristics, age was an important factor influencing health inequalities. Except for mental health, age exhibited a significant inverted U-shaped effect on the other three health dimensions (coefficients of the de-centered quadratic terms were all −0.001, *p* < 0.01, with turning points ranging from 42.22 to 66.52 years) (Figure 3). The impact of age on health inequalities reached its peak at these turning points and subsequently weakens with increasing age. This indicated that except for mental health, inequalities in other health dimensions peaked among middle-aged and young-older adult populations, while they were remaining lower among both younger and older age groups. The self-rated health and mental health inequalities of males were significantly higher than those of females (*β* = 0.364, *p* < 0.01). The impact of marital status was reflected in the significantly lower levels of self-rated health and mental health inequalities among single individuals compared to married individuals (*β* = −0.217 and − 0.268, respectively, *p* < 0.05). Household size was negatively correlated with self-rated health and two-week health inequalities (β = −0.035 and − 0.033, respectively, *p* < 0.01). The influence of ethnicity was not significant.

Regarding socioeconomic status characteristics, the PCHI had a negative impact on health inequalities, but this impact was more significant for self-rated health and mental health (*p* < 0.01) and not significant for two-week health and chronic disease status inequalities (*p* > 0.05). The urban group only exhibited a significant positive influence on chronic disease health inequalities (*β* = 0.138, *p* < 0.05). Years of education were positively correlated with mental health inequalities only at the 10% significance level (β = 0.027). The impact of agricultural occupation on health inequalities was not significant.

The effects of health behaviors (drinking, smoking, napping, exercise) and psychological attitudes (self-rated social status, life satisfaction) on health inequalities in various dimensions were not significant. Among the structural factors, the MIRR had no significant impact on health inequalities, but enrollment in basic medical insurance increased inequalities in chronic disease status (β = 0.067, *p* < 0.05). Lower level of medical expertise of the visited institutions also exacerbated inequalities in chronic disease status (β = 0.118, *p* < 0.01). Among the material circumstances factors, only the use of tap water had a significant negative impact on mental health and two-week health inequalities (*p* < 0.05).

### 3.4 Heterogeneity analysis of health inequalities within the China’s low-income population

The decomposition results in Tables S3–S5 revealed the heterogeneity of health inequality within the low-income population across urban–rural, gender, and age dimensions. The absolute values of WI regression coefficients were slightly higher than those of EI, but their direction, significance, and relative impact magnitudes remained consistent, demonstrating the robustness of the study’s findings. Given the high consistency between EI and WI results, subsequent analyses were based on the EI model results, with Tables S3–S5 simplified to Tables 5–7. Regarding urban–rural differences (Table 5), only two-week health inequalities exhibited significant total differences (*β* = 0.046, *p* < 0.05). The PCHI was the main source, manifesting as significant composition effects (−0.003, *p* < 0.05) and coefficient effects (−0.892, *p* < 0.05). Urban–rural differences in fuel use and the level of medical expertise of the visited institution significantly widened the between-group differences in urban–rural two-week health inequalities (composition effects of 0.024 and 0.003, respectively, *p* < 0.05). Although other health dimensions did not present significant total differences, the pure composition effects were significant for self-rated health and mental health, amounting to 0.018 (*p* < 0.01) and 0.024 (*p* < 0.05), respectively. Among them, the urban–rural difference in years of education significantly widened the between-group differences in urban–rural self-rated health and mental health inequalities, with pure composition effect coefficients of 0.007 (*p* < 0.05) and 0.010 (*p* < 0.05), respectively. The mean difference in age between urban and rural areas expanded the urban–rural disparity in mental health inequalities. The mean difference in the proportion of individuals visiting high-level medical institutions widened the urban–rural differences in self-rated health and two-week health inequalities. The urban–rural difference in the proportion of individuals using clean energy expanded the urban–rural disparities in self-rated health, mental health, and two-week health inequalities. The differences in the proportion of agricultural workers, those with low self-rated social status, and those with high life satisfaction primarily served to narrow the urban–rural disparities. Other factors had no significant impact on the urban–rural differences in health inequalities.

**Table 5:** RIF-Oaxaca decomposition of rural–urban health inequality (EI) in China, 2016–2022.

Variable	Self-rated health status	Mental health status	2-week health status	Chronic disease status
Rural	0.077*** (0.006)	0.055*** (0.008)	0.102*** (0.008)	0.040*** (0.007)
Counterfactual group	0.083*** (0.016)	0.064*** (0.021)	0.053** (0.022)	0.032* (0.019)
Urban	0.072*** (0.013)	0.054*** (0.018)	0.057*** (0.019)	0.039** (0.015)
Tdifference	0.005 (0.014)	0.002 (0.020)	0.044** (0.020)	0.001 (0.017)
ToT_explained	0.011 (0.012)	0.010 (0.015)	−0.005 (0.016)	−0.007 (0.014)
ToT_unexplained	−0.006 (0.022)	−0.008 (0.029)	0.049 (0.031)	0.007 (0.026)

	Explained	Unexplained	Explained	Unexplained	Explained	Unexplained	Explained	Unexplained
Total	0.011	−0.006	0.010	−0.008	−0.005	0.049	−0.007	0.007
Pure_explained	0.018**		0.024**		0.015		0.005	
Pure_unexplained		−0.001		−0.009		0.054*		0.008
Specif_err	−0.007		−0.014		−0.019**		−0.012	
Reweight_err		−0.005		<0.001		−0.005		−0.001

	Pure_explained	Pure_unexplained	Pure_explained	Pure_unexplained	Pure_explained	Pure_unexplained	Pure_explained	Pure_unexplained
LnPI	<0.001	0.023	<0.001	−0.024	0.001	−0.010	<0.001	0.010
LnPCHI	<0.001	−0.587	0.002	−0.616	−0.003**	−0.892**	<−0.001	−0.407
LnMIRR	0.001*	−0.010	<−0.001	0.020	0.001	−0.015	0.001	0.002
Age	0.004*	<0.001	0.006**	<0.001	0.003	0.001	0.001	0.001
Age^2^	0.001	−0.020	0.001	−0.022	0.001	−0.028	0.001	−0.004
Gender (reference group: female)								
Male	<−0.001	0.002	<0.001	−0.010	<0.001	−0.015	<0.001	−0.032
Ethnicity (reference group: other ethnicities)								
Han Chinese	−0.004	−0.031	0.005*	0.065	−0.003	−0.031	0.002	0.067
Marital status (reference group: married/cohabiting)								
Single	<0.001	0.014	<0.001	0.005	0.001	0.020	0.001	0.013
Divorced/Widowed	<0.001	0.004	0.001	0.003	−0.001	−0.009	0.001*	0.001
Years of education	0.007**	0.028	0.010**	0.076	0.001	0.020	0.002	0.001
Agricultural occupation (reference group: non-agricultural)								
Agricultural	−0.002	−0.022	−0.009	0.012	−0.007	−0.021	−0.013**	−0.014
Self-rated social status (reference group: average)								
Relatively low	−0.001	−0.004	−0.007***	−0.026	−0.003	−0.033	−0.001	−0.004
Relatively high	<0.001	0.005	0.003	−0.001	0.001	−0.007	−0.001	0.021
Past-month smoking (reference group: no)								
Yes	<0.001	−0.016	<−0.001	0.011	<−0.001	0.004	<−0.001	0.014

Variable	Self-rated health status		Mental health status		2-week health status		Chronic disease status	
	Pure_explained	Pure_unexplained	Pure_explained	Pure_unexplained	Pure_explained	Pure_unexplained	Pure_explained	Pure_unexplained
Frequent past-month alcohol use (reference group: no)								
Yes	−0.001*	0.013	<−0.001	−0.010	<−0.001	−0.007	<−0.001	−0.002
Nap habit (reference group: no)								
Yes	<−0.001	0.005	<−0.001	0.022	<0.001	−0.006	<−0.001	0.029
Weekly exercise (reference group: no)								
Yes	<−0.001	0.008	−0.002	0.006	<0.001	<−0.001	<−0.001	−0.001
Life satisfaction (reference group: somewhat satisfied)								
Dissatisfied/Neutral	−0.001	0.013	<0.001	0.020	<−0.001	−0.003	0.002**	0.027
Very satisfied	<−0.001	0.031	−0.001	0.014	−0.001	0.029	−0.004***	0.031
Cooking water source (reference group: non-tap water)								
Tap water	<0.001	0.012	<−0.001	−0.064	−0.003	−0.024	0.003	0.009
Cooking fuel type (reference group: non-clean fuels)								
Clean fuels	0.010**	0.006	0.015**	−0.003	0.024***	0.038	0.008	−0.007
Household size	<0.001	−0.034	<0.001	0.010	<0.001	−0.012	<0.001	−0.007
Basic social medical insurance enrollment (reference group: not enrolled)								
Enrolled	<0.001	0.051	−0.002	0.085	−0.001	0.028	0.001	0.028
Level of medical expertise of the visited institution (reference group: fair)								
Low	<0.001	−0.001	<−0.001	0.012	<−0.001	0.013	0.001	−0.004
High	0.003***	−0.013	0.001	0.004	0.003***	−0.019	0.001	−0.005
Year FE	Yes	Yes	Yes	Yes	Yes	Yes	Yes	Yes

*, **, and *** denote significance at the 10, 5, and 1% levels, respectively. PI, Personal Income; PCHI, Per Capita Household Income; MIRR, Medical Insurance Reimbursement Ratio. The complete table is available in Table S3; only variables and their categories with statistically significant estimated coefficients are presented here.

**Table 6:** RIF-Oaxaca decomposition of male–female health inequality (EI) in China, 2016–2022.

Variable	Self-rated health status	Mental health status	2-week health status	Chronic disease status
Female	0.066*** (0.008)	0.051*** (0.011)	0.091*** (0.012)	0.029*** (0.010)
Counterfactual group	0.068*** (0.025)	0.054** (0.027)	0.070** (0.029)	0.112*** (0.032)
Male	0.079*** (0.008)	0.065*** (0.011)	0.074*** (0.012)	0.047*** (0.009)
Tdifference	−0.012 (0.011)	−0.013 (0.016)	0.017 (0.016)	−0.018 (0.013)
ToT_explained	−0.010 (0.022)	−0.011 (0.023)	−0.004 (0.026)	0.065** (0.029)
ToT_unexplained	−0.002 (0.026)	−0.002 (0.029)	0.021 (0.032)	−0.083** (0.034)

	Explained	Unexplained	Explained	Unexplained	Explained	Unexplained	Explained	Unexplained
Total	−0.010	−0.002	−0.011	−0.002	−0.004	0.021	0.065**	−0.083**
Pure_explained	−0.005		0.001		−0.009		0.004	
Pure_unexplained		0.005		−0.010		0.022		−0.081***
Specif_err	−0.005		−0.012		0.004		0.061***	
Reweight_err		−0.007		0.007		<−0.001		−0.002

	Pure_explained	Pure_unexplained	Pure_explained	Pure_unexplained	Pure_explained	Pure_unexplained	Pure_explained	Pure_unexplained
LnPI	−0.002	0.022	−0.004	−0.034	−0.002	0.029	−0.003	0.088
LnPCHI	0.003**	0.195	0.008**	0.884*	−0.001	0.100	<−0.001	−0.640**
LnMIRR	0.001	0.014	<−0.001	0.037	0.002	0.086	0.001	−0.084
Age	−0.001*	−0.001	−0.003**	<−0.001	−0.002*	0.001	−0.001	−0.001
Age^2^	<0.001	−0.026	<0.001	−0.004	<0.001	0.024	<0.001	0.019
Residence (reference group: rural)								
Urban	<−0.001	−0.016	<0.001	−0.006	−0.001	−0.012	<−0.001	−0.051*
Ethnicity (reference group: other ethnicities)								
Han Chinese	<−0.001	−0.027	<−0.001	0.028	<−0.001	−0.022	<−0.001	−0.115**
Marital status (reference group: married/cohabiting)								
Single	0.001	0.011	<0.001	−0.004	<0.001	−0.036**	0.001	−0.005
Divorced/Widowed	−0.001	−0.001	−0.005	0.034**	0.002	−0.007	−0.006	−0.007
Years of education	−0.002	0.014	−0.003	−0.028	−0.008*	0.007	<−0.001	0.105**
Agricultural occupation (reference group: non-agricultural)								
Agricultural	−0.001	−0.003	−0.001	0.067**	−0.001	−0.032	−0.001	−0.032
Self-rated social status (reference group: average)								
Relatively low	<0.001	−0.022	0.001	−0.016	<−0.001	0.037*	<0.001	−0.017
Relatively high	<0.001	−0.016	0.001	−0.017	<0.001	0.009	<0.001	0.001
Past-month smoking (reference group: no)								
Yes	−0.008	−0.002	0.006	−0.002	−0.007	−0.001	0.005	0.002

Variable	Self-rated health status		Mental health status		2-week health status		Chronic disease status	
	Pure_explained	Pure_unexplained	Pure_explained	Pure_unexplained	Pure_explained	Pure_unexplained	Pure_explained	Pure_unexplained
Frequent past-month alcohol use (reference group: no)								
Yes	0.001	−0.001	0.003	0.002	0.006	0.002	0.007	0.003
Nap habit (reference group: no)								
Yes	<0.001	−0.013	<0.001	0.017	0.001	−0.016	0.001	−0.083**
Weekly exercise (reference group: no)								
Yes	<0.001	0.009	<−0.001	0.010	0.001	0.034*	0.001	0.042**
Life satisfaction (reference group: somewhat satisfied)								
Dissatisfied/Neutral	<0.001	0.008	<−0.001	0.041*	<−0.001	0.040	<−0.001	−0.005
Very satisfied	<0.001	−0.012	−0.001	0.017	−0.001	−0.016	−0.001	<0.001
Cooking water source (reference group: non-tap water)								
Tap water	<−0.001	0.015	<−0.001	0.060	<−0.001	0.013	<−0.001	0.013
Cooking fuel type (reference group: non-clean fuels)								
Clean fuels	<−0.001	−0.013	<−0.001	−0.058	<0.001	−0.027	<−0.001	−0.002
Household size	0.002	−0.028	0.001	−0.002	0.001	−0.103*	0.001	−0.070
Basic social medical insurance enrollment (reference group: not enrolled)								
Enrolled	<−0.001	−0.123*	<0.001	−0.050	<0.001	0.166	−0.001	−0.160*
Level of medical expertise of the visited institution (reference group: fair)								
Low	−0.001	−0.003	−0.001	−0.007	−0.001	0.007	−0.003***	−0.004
High	0.004***	−0.056	<0.001	−0.015	0.003	<−0.001	0.004**	−0.046
Year FE	Yes	Yes	Yes	Yes	Yes	Yes	Yes	Yes

*, **, and *** denote significance at the 10, 5, and 1% levels, respectively. PI, Personal Income; PCHI, Per Capita Household Income; MIRR, Medical Insurance Reimbursement Ratio. The complete table is available in Table S4; only variables and their categories with statistically significant estimated coefficients are presented here.

**Table 7:** RIF-Oaxaca decomposition of age-related health inequality (EI) in China, 2016–2022.

Variable	Self-rated health status	Mental health status	2-week health status	Chronic disease status
Age < 60	0.024*** (0.007)	0.020** (0.010)	0.037*** (0.010)	−0.003 (0.007)
Counterfactual group	−0.023 (0.022)	0.092*** (0.035)	0.025 (0.040)	−0.038 (0.040)
Age ≥ 60	0.073*** (0.009)	0.101*** (0.014)	0.078*** (0.014)	0.014 (0.013)
Tdifference	−0.049*** (0.011)	−0.081*** (0.017)	−0.041** (0.017)	−0.017 (0.015)
ToT_explained	−0.095*** (0.021)	−0.009 (0.033)	−0.053 (0.038)	−0.052 (0.038)
ToT_unexplained	0.047* (0.024)	−0.072* (0.038)	0.012 (0.044)	0.035 (0.044)

	Explained	Unexplained	Explained	Unexplained	Explained	Unexplained	Explained	Unexplained
Total	−0.095***	0.047*	−0.009	−0.072*	−0.053	0.012	−0.052	0.035
Pure_explained	−0.020**		0.020		0.007		−0.010	
Pure_unexplained		0.054		−0.012		0.055		0.071
Specif_err	−0.075***		−0.029		−0.060*		−0.041	
Reweight_err		−0.008		−0.059		−0.043		−0.036

	Pure_explained	Pure_unexplained	Pure_explained	Pure_unexplained	Pure_explained	Pure_unexplained	Pure_explained	Pure_unexplained
lnPI	0.002	0.011	0.008*	−0.020	0.004	0.046	−0.019***	0.127*
lnPCHI	−0.005***	0.311	−0.007**	0.017	0.009***	−0.134	<−0.001	−0.054
LnMIRR	−0.001	−0.029	0.003	0.047	0.004	−0.041	−0.001	−0.015
Gender (reference group: female)								
Male	<−0.001	−0.017	<−0.001	−0.005	<−0.001	−0.056	<−0.001	−0.016
Residence (reference group: rural)								
Urban	<0.001	0.007	0.003**	−0.007	−0.001	0.020	−0.003*	0.028
Ethnicity (reference group: other ethnicities)								
Han Chinese	−0.006***	−0.124*	0.006*	0.027	−0.005	−0.106	<−0.001	0.118
Marital status (reference group: married/cohabiting)								
Single	−0.006***	0.019	−0.007**	0.102**	−0.005*	0.077	−0.001	0.043
Divorced/Widowed	0.007**	0.001	0.004	0.003	−0.005	−0.010	0.006	0.007
Years of education	0.006	0.033	0.019**	−0.095	0.023***	−0.018	0.008	0.059
Agricultural occupation (reference group: non-agricultural)								
Agricultural	0.005***	0.035*	0.007***	0.055*	0.004**	0.015	0.004**	−0.002
Self-rated social status (reference group: average)								
Relatively low	0.002	0.013	0.013***	−0.013	0.005	0.018	−0.005*	0.044
Relatively high	−0.009***	−0.023*	−0.009**	−0.002	−0.004	−0.003	0.003	0.010
Past-month smoking (reference group: no)								
Yes	<0.001	−0.009	<0.001	0.016	<0.001	0.015	<0.001	−0.018

Variable	Self-rated health status		Mental health status		2-week health status		Chronic disease status	
	Pure_explained	Pure_unexplained	Pure_explained	Pure_unexplained	Pure_explained	Pure_unexplained	Pure_explained	Pure_unexplained
Frequent past-month alcohol use (reference group: no)								
Yes	<−0.001	−0.001	<0.001	−0.001	<0.001	0.015	<−0.001	−0.008
Nap habit (reference group: no)								
Yes	−0.002	−0.003	−0.002	0.053	−0.010***	0.030	−0.010***	−0.025
Weekly exercise (reference group: no)								
Yes	−0.001	0.008	−0.004***	−0.009	−0.002	−0.010	−0.001	0.005
Life satisfaction (reference group: somewhat satisfied)								
Dissatisfied/Neutral	0.004	−0.010	0.006	−0.010	0.002	−0.005	0.003	−0.074
Very satisfied	<0.001	−0.001	−0.001	0.011	0.008**	0.009	0.007**	−0.036
Cooking water source (reference group: non-tap water)								
Tap water	<−0.001	0.015	−0.003**	0.081	−0.001	0.036	<−0.001	0.027
Cooking fuel type (reference group: non-clean fuels)								
Clean fuels	0.001	−0.023	−0.001	−0.027	−0.002	0.017	<−0.001	−0.072
Household size	−0.013***	0.022	−0.014**	0.072	−0.013**	0.006	−0.001	0.077
Basic social medical insurance enrollment (reference group: not enrolled)								
Enrolled	−0.001	−0.096	<0.001	−0.081	−0.001	−0.028	−0.002*	−0.064
Level of medical expertise of the visited institution (reference group: fair)								
Low	<0.001	0.004	0.003*	−0.006	0.001	−0.020	0.004**	−0.016
High	−0.005***	0.003	−0.005*	−0.018	−0.006**	−0.010	−0.001	0.029
Year FE	Yes	Yes	Yes	Yes	Yes	Yes	Yes	Yes

*, **, and *** denote significance at the 10, 5, and 1% levels, respectively. PI Personal Income. PCHI Per Capita Household Income. MIRR Medical Insurance Reimbursement Ratio. The complete table is available in Table S5; only variables and their categories with statistically significant estimated coefficients are presented here.

The gender difference analysis showed no significant total differences in any health dimension (Table 6). However, the PCHI remained a significant source of gender differences in health inequalities across multiple dimensions: the pure composition effects on gender differences in self-rated health and mental health inequalities were 0.003 (*p* < 0.05) and 0.008 (*p* < 0.05), respectively; the pure coefficient effect on chronic disease health inequalities was −0.64 (*p* < 0.05). The between-group mean difference in age narrowed the gender difference in mental health inequalities. The gender difference in the proportion of individuals visiting high-level medical institutions widened the gender differences in self-rated health and chronic disease status inequalities. The impact of marital status differs between males and females: being divorced, widowed, or engaged in agriculture had significant positive coefficient effects on the gender differences in mental health inequalities. The PCHI, ethnicity, napping, and being single had negative coefficient effects on the gender differences in health inequalities. Years of education and exercise had positive coefficient effects on the gender differences in chronic disease status inequalities. Other factors had no significant impact on the gender differences in health inequalities.

Regarding age group differences (Table 7), significant inequalities existed in self-rated health, mental health, and two-week health, with total difference effects of −0.049 (*p* < 0.01), −0.081 (*p* < 0.01), and − 0.041 (*p* < 0.05), respectively. Among them, the composition effect of self-rated health inequality was −0.095 (*p* < 0.01), accounting for 194% of the total difference effect. The between-group difference in the PCHI had varying impacts on different health dimensions: its pure composition effected on self-rated health and mental health inequalities were − 0.005 and − 0.007 (both *p* < 0.01), respectively, while it was 0.009 (*p* < 0.01) for two-week health inequality. The between-group difference in the PI narrowed the between-group difference in chronic disease status inequality (pure composition effect of −0.019, *p* < 0.01). The between-group difference in years of education widened the between-group differences in mental health and two-week health inequalities; the difference in the proportion of agricultural workers expanded the between-group differences in health inequalities across all dimensions; while the difference in household size narrowed the between-group differences in self-rated health, mental health, and two-week health inequalities. The difference in the proportion of the urban population expanded the between-group difference in mental health inequality. The difference in the proportion of Han Chinese narrowed the between-group difference in self-rated health inequality. The between-group differences in marital status primarily affected the between-group differences in self-rated health and mental health inequalities. Self-rated social status mainly influenced the between-group differences in self-rated health and mental health inequalities. Life satisfaction and napping primarily affected the between-group differences in two-week health and chronic disease status inequalities. Exercise and cooking water affected the between-group difference in mental health inequality. The differences in the level of medical institutions significantly impacted the between-group differences in self-rated health, two-week health, and chronic disease status inequalities. Other factors had no significant influence on the age group differences in health inequalities.

## 4 Discussion

This study revealed that China’s low-income population faces severe health inequality. Although the upper limit of the PCHI for the low-income population increased annually from 2010 to 2022, the widening gap between the rich and the poor kept them in a disadvantaged position (63). From 2018 to 2022, influenced by factors such as the COVID-19 pandemic and changes in the global economic situation, the growth rate of the PCHI among low-income population slowed down. Despite a slight recovery during 2020–2022, the overall growth rate remained lower than pre-2018 levels. Meanwhile, the proportion of healthy individuals in terms of two-week health status showed a U-shaped pattern (with 2018 as the turning point), indicating that low-income population’s health vulnerability became more pronounced when facing both sluggish income growth and pandemic-related impacts. The absolute values of health inequality indices among the low-income group were generally higher than those of the middle-to-high-income group during the same period, which was consistent with international findings (11, 64–66), especially in developing countries (6). This indicated that, compared to the middle-to-high-income group, the low-income group faced more severe health inequalities. Additionally, health inequalities among the low-income population were also quite serious (6). During the study period, the low-income population exhibited pro-rich inequalities across all four health dimensions, with the inequality levels in self-rated health and chronic disease dimensions increasing year by year, reflecting a continuous deterioration of health inequalities (67). Therefore, addressing health inequalities in China requires a focus on the low-income population, particularly the impoverished individuals within this population.

Although health inequalities within the low-income population did not show significant differences between urban and rural areas (6), genders, or age groups, pro-rich health inequalities were prevalent in all subgroups. Consistent with previous research (12, 24), we found that from 2012 to 2022, the self-rated health inequality index of the rural low-income group showed an upward trend and remained consistently higher than that of urban group since 2014. This may be related to the uneven distribution of medical resources between urban and rural areas (68). According to China’s national statistics, the country’s urbanization rate steadily increased from 53.10 to 65.22% between 2010 and 2022, without experiencing drastic fluctuations. This suggests that changes in urban–rural health inequalities were likely more influenced by the distribution of medical resources and economic factors rather than the urbanization process itself. Further examination of CFPS data revealed that the proportion of the urban population in the study sample remained stable between 2010 and 2022, ranging from 41.7 to 42.5%, aligning with the national trend. This indicates that the data in this study possessed a representative urban–rural structure. Regarding the trend of health inequalities, it is noteworthy that since 2020, the significance of self-rated health and mental health inequalities in urban areas declined significantly compared to 2018 and earlier, with the indices in 2020 becoming non-significant. This may have been associated with urban residents receiving more comprehensive psychological assistance and social support systems during the COVID-19 pandemic. Similarly, this effect weakened in 2022, suggesting a declining marginal effect of pandemic interventions and policy benefits. In contrast, the older adult low-income group exhibited significantly higher levels of inequality in self-rated health, mental health, and two-week health compared to the non-older adult group (69), with the inequality index being most significant in the mental health dimension, and the significance of mental health dimension appeared unaffected by the COVID-19 pandemic. The older adult low-income group, generally more vulnerable in terms of economic and social support networks, did not receive sufficient resources during the pandemic to significantly improve their mental health status, resulting in persistently high levels of inequality in this dimension. Conversely, for urban areas or younger groups, relatively adequate social and psychological support measures may have served as a buffer (70). These findings emphasize the need for targeted interventions for rural (19) and older adult low-income groups, particularly regarding their mental health. Furthermore, to further explore the interaction effects among urban–rural status, gender, and age, we included interaction terms in the RIF regression models. However, the results were not statistically significant overall, indicating that these factors primarily influenced health inequalities through direct effects rather than interactive mechanisms.

The PCHI was a key socioeconomic factor influencing health inequalities among and within the low-income population. The RIF regression results showed that improvements in household economic conditions could significantly reduce inequalities in self-rated health and mental health. This aligned with previous research conclusions, as income level is widely recognized as an important determinant of health outcomes (6, 13, 15). Further RIF-Oaxaca decomposition results revealed that the PCHI was a significant source of health inequality differences between urban and rural areas, genders, and age groups. Its function was primarily reflected in two aspects: first, the mean differences in the PCHI between different groups (urban–rural, gender, age) directly led to health inequalities; second, even when holding the PCHI constant, this variable still contributed to the between-group differences in health inequalities across urban and rural groups, suggesting that PCHI may interact with other factors to influence the urban–rural difference in health inequalities. This may be because urban–rural differences primarily stemmed from the urban–rural inequality in the accessibility of medical resources. Even with the same income level, the rural group still had fewer opportunities to obtain high-quality medical services compared to the urban group (68). Gender differences may be related to women’s weaker bargaining power in household income allocation, resulting in a lower effect of the same household income on improving women’s health. Family support is significantly associated with women’s health (71). Furthermore, agricultural occupation affected health inequalities through gender differences, particularly in mental health and two-week health. This may be due to the heavy burden on rural women’s physical and mental health as they undertook both strenuous agricultural labor and household chores (72).

Years of education had no significant impact on total health inequalities among the low-income population, which contradicts previous research (73). This discrepancy may be attributed to the focus of this study on the low-income population, which generally had lower educational attainment (84.22% had junior secondary school or below), thus limiting the positive influence of education on health. However, the decomposition results revealed that years of education still had significant effects on specific subgroups. The mean differences in years of education between urban and rural groups affected the urban–rural disparities in self-rated health and mental health inequalities, possibly due to variations in educational quality and access to educational resources between urban and rural regions (74). Regarding age group differences, the between-group mean differences in years of education influenced the between-group differences in mental health and two-week health inequalities. This may be because educational content and its social value have changed over time. Individuals with the same years of education may possess different knowledge and skills (75). Moreover, with the popularization of higher education in China (76), the social status associated with the same educational level has also shifted across generations. Gender differences caused by education may be related to gender disparities in role norms and occupational choices (72), which in turn affect chronic disease prevention and management behaviors.

Sociodemographic characteristics were another critical dimension influencing health inequalities among and within the low-income population. Age had the most significant impact, exhibiting an inverted U-shaped relationship: as age increased, health inequalities first increased and then decreased. This finding is consistent with a study conducted in South Korea (77). According to the Cumulative Advantage/Disadvantage theory, health capital may also accumulate with age, thereby exacerbating health inequalities (78). However, after a certain age, the health status of the older adult generally declines, thus alleviating health inequalities. The impact of marital status exhibited gender and age heterogeneity. Compared to married or cohabiting individuals, single individuals had lower levels of self-rated health and mental health inequalities. The effects of divorce/widowhood on health inequalities also differed between men and women and across age groups. This may be related to the social roles and expectations associated with different genders and ages. It also suggests that marital patterns themselves, rather than marital status, may have a greater impact on mental health. Household size had a universal suppressive effect on health inequalities, particularly in reducing disparities between age groups, which may be related to the informal care and economic risk-sharing functions provided by larger household sizes (22).

Among structural factors, the impact of healthcare accessibility surpassed that of health insurance coverage, possibly due to the current high insurance participation rate (over 90%) among China’s low-income population. Consistent with previous research (79), disparities in the level of medical expertise of the visited institutions significantly influenced health inequalities in chronic diseases status and played a crucial role in urban–rural, gender, and age group differences. This finding suggests that the uneven distribution of quality healthcare resources might be a key factor exacerbating health inequalities (68). The effects of health behaviors, material circumstances, and psychological attitudes were relatively limited. This may be due to the strong homogeneity in health behaviors among the low-income population: economic constraints limit their choices for health investments, resulting in smaller differences in health behaviors. Among material circumstances, only access to tap water, an infrastructure indicator, was found to significantly reduce mental health and two-week health inequalities. The impact of other material circumstances was limited, possibly because the current differences in living environment conditions among the low-income population are relatively small. Psychological attitude variables mainly affected health inequalities through age group heterogeneity, possibly explained by generational differences in psychological resilience and coping mechanisms across different age groups.

Based on the above analysis, this study proposes the following policy recommendations to alleviate health inequalities for the low-income population. In the short term, emphasis should be placed on income support and healthcare service accessibility. Regarding income support, it is recommended to establish minimum living security standards for low-income older adult individuals, increase rural pension benefits, and provide subsidies for daily necessities and medical expense reductions for multiply disadvantaged groups such as older adult rural women. Additionally, family income sources should be expanded through industrial assistance programs. In terms of healthcare services, efforts should focus on training general practitioners and implementing systems like “county management, township employment” to guide quality resources to grassroots levels, improving the stability of primary healthcare professionals. Meanwhile, artificial intelligence-assisted diagnostic systems and internet healthcare platforms should be fully utilized to provide remote consultation and intelligent screening support for primary healthcare institutions (80–83), enhancing the accessibility and quality of basic medical services. In the medium to long term, improvements should focus on family support systems and optimization of medical resource allocation. Considering the significant suppressive effect of household size on health inequalities, it is recommended to establish a family caregiver allowance system, develop community-based older adult care services, and provide differentiated social support through neighborhood mutual assistance networks for special groups such as single, divorced, and widowed individuals. To address the uneven distribution of medical resources, systems should be established for targeted training and rotation of urban–rural medical professionals, promotion of telemedicine services, and strengthening primary healthcare institutions’ capabilities in chronic disease management and mental health services. Simultaneously, health literacy improvement projects should be conducted for low-income groups, with community health management teams providing personalized health guidance. To address barriers in accessing health information caused by inadequate infrastructure and educational resources in rural areas, investment in public infrastructure and education should be increased to promote the equalization of basic public services. Furthermore, a monitoring and intervention mechanism for health inequalities should be established, including a scientific evaluation indicator system to regularly assess changes in health disparities among various groups. The implementation of these intervention measures should adopt a progressive approach, prioritizing regions and populations with higher levels of health inequalities, while strengthening the evaluation of policy implementation effects to provide empirical evidence for subsequent policy optimization.

This study still has some limitations. First, due to the long survey period of the CFPS dataset and the considerable missing data for some key variables (e.g., social capital and early-life factors), our ability to fully analyze the mechanisms influencing health inequalities is limited. Additionally, although this study controlled for temporal and regional factors, it was still unable to directly measure the impact of individual-level migration on health inequalities. Panel data were also affected by individual migration, mortality, refusal, or loss to follow-up, which may have led to sample attrition, potentially impacting the representativeness of the conclusions. Moreover, due to the limited number of repeated individual observations, this study did not employ a dynamic panel model, which would have allowed for a more precise depiction of the long-term evolution of health inequalities. Furthermore, while the data span 2010–2022, the dynamic adjustments and prolonged impact of COVID-19 control policies introduce uncertainties, making it challenging to comprehensively assess the pandemic’s long-term effects on health inequalities. Future research should integrate multi-source data (including macroeconomic indicators) and adopt dynamic panel models and instrumental variable methods to further investigate the long-term effects of migration, pandemic shocks, and other socioeconomic factors on health inequalities. These efforts will provide stronger empirical evidence to support policies aimed at promoting health equity.

## 5 Conclusion

Based on CFPS data from 2010–2022, this study employed various methods such as the EI, WI, RIF regression, and RIF-Oaxaca decomposition to systematically analyze the health inequalities faced by China’s low-income population. The study found that the health inequalities faced by the low-income population are not only severe but also exhibit an aggravating trend. Across all health dimensions, the low-income group demonstrated significant pro-rich inequalities, with levels far exceeding those of the middle-to-high-income group. The PCHI is the core influencing factor of health inequalities, not only directly affecting the inequalities of self-rated health and mental health but also serving as the main source of between-group differences in health inequalities across urban–rural, genders, and age groups. In particular, the older adult group and rural group face the most severe health inequalities. Based on the research findings, this paper proposes a phased policy intervention framework. In the short term, targeted income support policies should be implemented, focusing on intersectional disadvantaged groups such as the rural older adult, while simultaneously improving the accessibility and quality of primary healthcare services through healthcare reforms. Long-term strategies should focus on leveraging the protective effect of household size on health inequalities, establishing a support system that considers the differentiated impact of marital status on different genders and age groups, and increasing investment in infrastructure in rural areas. These intervention measures should be implemented progressively, prioritizing regions and groups with higher levels of health inequalities.

## Data Availability

Publicly available datasets were analyzed in this study. This data can be found at: http://www.isss.pku.edu.cn/cfps/index.htm.
