# Supplementary material for: Health inequalities for China’s low-income population: trends, subgroup differences, and influencing factors, 2010–2022
Source: Front Public Health. 2025 Apr 10;13:1569726. doi: 10.3389/fpubh.2025.1569726 (PMC12018423; doi:10.3389/fpubh.2025.1569726)
Supplement: Supplementary file 1 [file Data_Sheet_1.DOCX]

Supplementary Material

**Table S1** Health status and trends of national residents in China, 2010-2022

| **Variable** | **2010** | | **2012** | | **2014** | | **2016** | | **2018** | | **2020** | | **2022** | |
| --- | --- | --- | --- | --- | --- | --- | --- | --- | --- | --- | --- | --- | --- | --- |
|  | **N/mean** | **%/SE** | **N/mean** | **%/SE** | **N/mean** | **%/SE** | **N/mean** | **%/SE** | **N/mean** | **%/SE** | **N/mean** | **%/SE** | **N/mean** | **%/SE** |
| **Self-rated Health Status** | | | | | | | | | | | | | | |
| Unhealthy | 506 | 1.62 | 4,660 | 15.83 | 3,971 | 13.62 | 4,748 | 15.98 | 3,905 | 15.13 | 2,551 | 12.78 | 2,418 | 13.30 |
| Average | 2,335 | 7.49 | 5,386 | 18.29 | 4,209 | 14.43 | 5,817 | 19.59 | 3,294 | 12.76 | 2,025 | 10.14 | 1,575 | 8.67 |
| Relatively healthy | 1,638 | 5.25 | 10,264 | 34.86 | 10,288 | 35.28 | 10,484 | 35.30 | 11,008 | 42.66 | 8,901 | 44.58 | 8,459 | 46.54 |
| Very healthy | 10,821 | 34.71 | 6,245 | 21.21 | 6,339 | 21.74 | 5,087 | 17.13 | 4,162 | 16.13 | 3,375 | 16.90 | 2,953 | 16.25 |
| Extremely healthy | 15,873 | 50.92 | 2,889 | 9.81 | 4,351 | 14.92 | 3,567 | 12.01 | 3,435 | 13.31 | 3,114 | 15.60 | 2,771 | 15.24 |
| **Mental health status** | | | | | | | | | | | | | | |
| Unhealthy | 7,379 | 23.67 | 4,816 | 16.36 | 7,657 | 26.26 | 5,414 | 18.23 | 5,379 | 20.85 | 4,236 | 21.21 | 4,298 | 23.65 |
| Healthy | 23,794 | 76.33 | 24,628 | 83.64 | 21,501 | 73.74 | 24,289 | 81.77 | 20,424 | 79.15 | 15,731 | 78.79 | 13,878 | 76.35 |
| **2-week health status** | | | | | | | | | | | | | | |
| Healthy | 23,726 | 76.11 | 21,051 | 71.50 | 20,749 | 71.16 | 20,719 | 69.75 | 17,916 | 69.43 | 15,030 | 75.28 | 13,422 | 73.85 |
| Unhealthy | 7,447 | 23.89 | 8,393 | 28.50 | 8,409 | 28.84 | 8,984 | 30.25 | 7,887 | 30.57 | 4,937 | 24.72 | 4,753 | 26.15 |
| **Chronic disease status** | | | | | | | | | | | | | | |
| Without chronic diseases | 27,355 | 87.75 | 26,214 | 89.03 | 24,768 | 84.94 | 24,184 | 81.42 | 21,939 | 85.02 | 17,277 | 86.53 | 15,371 | 84.57 |
| With chronic disease | 3,818 | 12.25 | 3,230 | 10.97 | 4,390 | 15.06 | 5,519 | 18.58 | 3,864 | 14.98 | 2,690 | 13.47 | 2,804 | 15.43 |
| **Total** | 31,173 |  | 29,444 |  | 29,158 |  | 29,703 |  | 25,803 |  | 19,967 |  | 18,176 | |

*SE* Standard Error.

**Table S2** Health inequality indices within low-income population in China, 2010-2022

| **Health variables** | **Year** | **Rural** | | **Urban** | | **Female** | | **Male** | | **Age<60** | | **Age≥60** | |
| --- | --- | --- | --- | --- | --- | --- | --- | --- | --- | --- | --- | --- | --- |
|  |  | **WI** | **EI** | **WI** | **EI** | **WI** | **EI** | **WI** | **EI** | **WI** | **EI** | **WI** | **EI** |
| **Self-rated health status** | 2022 | 0.076*** | 0.076*** | 0.059* | 0.059* | 0.073*** | 0.073*** | 0.063*** | 0.063*** | 0.034 | 0.033 | 0.061** | 0.058** |
|  |  | (0.019) | (0.019) | (0.026) | (0.026) | (0.020) | (0.020) | (0.019) | (0.018) | (0.017) | (0.017) | (0.021) | (0.020) |
|  | z-value | -0.53 | -0.53 |  |  | -0.37 | -0.37 |  |  | 1 | 0.94 |  |  |
|  | 2020 | 0.070*** | 0.070*** | 0.040 | 0.040 | 0.045* | 0.045* | 0.070*** | 0.069*** | 0.012 | 0.012 | 0.074*** | 0.070*** |
|  |  | (0.018) | (0.018) | (0.020) | (0.020) | (0.018) | (0.018) | (0.018) | (0.018) | (0.016) | (0.016) | (0.018) | (0.017) |
|  | z-value | -1.07 | -1.08 |  |  | 0.96 | 0.95 |  |  | 2.530* | 2.470* |  |  |
|  | 2018 | 0.072*** | 0.071*** | 0.070*** | 0.070*** | 0.072** | 0.071** | 0.070**** | 0.070**** | 0.044** | 0.044** | 0.041 | 0.037 |
|  |  | (0.021) | (0.020) | (0.020) | (0.020) | (0.023) | (0.022) | (0.015) | (0.015) | (0.017) | (0.017) | (0.022) | (0.020) |
|  | z-value | -0.05 | -0.05 |  |  | -0.1 | -0.07 |  |  | -0.13 | -0.27 |  |  |
|  | 2016 | 0.069** | 0.069** | 0.064*** | 0.064*** | 0.064*** | 0.063*** | 0.065*** | 0.065*** | 0.024 | 0.024 | 0.052** | 0.046** |
|  |  | (0.021) | (0.021) | (0.018) | (0.018) | (0.019) | (0.019) | (0.019) | (0.019) | (0.018) | (0.018) | (0.019) | (0.017) |
|  | z-value | -0.17 | -0.19 |  |  | 0.05 | 0.1 |  |  | 1.04 | 0.89 |  |  |
|  | 2014 | 0.037 | 0.037 | 0.026 | 0.026 | 0.025 | 0.024 | 0.042** | 0.042** | 0.012 | 0.012 | 0.037 | 0.034 |
|  |  | (0.020) | (0.020) | (0.019) | (0.019) | (0.016) | (0.016) | (0.016) | (0.016) | (0.013) | (0.013) | (0.020) | (0.018) |
|  | z-value | -0.420 | -0.420 |  |  | 0.780 | 0.780 |  |  | 1.040 | 0.970 |  |  |
|  | 2012 | 0.018 | 0.018 | 0.055* | 0.055* | 0.028 | 0.028 | 0.043 | 0.043 | 0.006 | 0.006 | 0.030 | 0.024 |
|  |  | (0.034) | (0.034) | (0.022) | (0.021) | (0.028) | (0.028) | (0.022) | (0.022) | (0.023) | (0.023) | (0.027) | (0.023) |
|  | z-value | 0.920 | 0.940 |  |  | 0.410 | 0.430 |  |  | 0.660 | 0.570 |  |  |
|  | 2010 | 0.070** | 0.051** | 0.085*** | 0.054*** | 0.074*** | 0.055*** | 0.091**** | 0.060**** | 0.063** | 0.039** | 0.018 | 0.016 |
|  |  | (0.022) | (0.016) | (0.025) | (0.016) | (0.020) | (0.014) | (0.021) | (0.014) | (0.021) | (0.013) | (0.022) | (0.020) |
|  | z-value | 0.470 | 0.130 |  |  | 0.590 | 0.280 |  |  | -1.510 | -0.960 |  |  |
| **Mental health status** | 2022 | 0.074* | 0.062* | 0.110* | 0.087* | 0.083* | 0.073* | 0.096** | 0.071** | 0.059 | 0.047 | 0.134*** | 0.114*** |
|  |  | (0.032) | (0.026) | (0.051) | (0.041) | (0.032) | (0.028) | (0.032) | (0.024) | (0.035) | (0.028) | (0.038) | (0.032) |
|  | z-value | 0.6 | 0.53 |  |  | 0.29 | -0.06 |  |  | 1.47 | 1.59 |  |  |
|  | 2020 | 0.074* | 0.061* | 0.065 | 0.049 | 0.078* | 0.067* | 0.069* | 0.051* | 0.035 | 0.027 | 0.118** | 0.102** |
|  |  | (0.029) | (0.024) | (0.044) | (0.033) | (0.032) | (0.027) | (0.031) | (0.023) | (0.030) | (0.023) | (0.038) | (0.033) |
|  | z-value | -0.16 | -0.29 |  |  | -0.21 | -0.45 |  |  | 1.72 | 1.87 |  |  |
|  | 2018 | 0.106*** | 0.086**** | 0.116*** | 0.085*** | 0.110**** | 0.092**** | 0.123**** | 0.087**** | 0.101*** | 0.074*** | 0.107*** | 0.092*** |
|  |  | (0.023) | (0.019) | (0.028) | (0.021) | (0.020) | (0.017) | (0.029) | (0.020) | (0.025) | (0.018) | (0.026) | (0.023) |
|  | z-value | 0.29 | -0.01 |  |  | 0.37 | -0.18 |  |  | 0.15 | 0.62 |  |  |

**Table S2** (Continued).

| **Health variables** | **Year** | **Rural** | | **Urban** | | **Female** | | **Male** | | **Age<60** | | **Age≥60** | |
| --- | --- | --- | --- | --- | --- | --- | --- | --- | --- | --- | --- | --- | --- |
|  |  | **WI** | **EI** | **WI** | **EI** | **WI** | **EI** | **WI** | **EI** | **WI** | **EI** | **WI** | **EI** |
| **Mental health status** | 2016 | 0.113** | 0.085** | 0.179**** | 0.127**** | 0.120**** | 0.097**** | 0.182**** | 0.116**** | 0.097** | 0.064** | 0.170**** | 0.140**** |
|  |  | (0.036) | (0.027) | (0.035) | (0.025) | (0.028) | (0.023) | (0.041) | (0.026) | (0.036) | (0.024) | (0.031) | (0.025) |
|  | z-value | 1.3 | 1.15 |  |  | 1.23 | 0.57 |  |  | 1.55 | 2.2 |  |  |
|  | 2014 | 0.045 | 0.010 | 0.113 | 0.017 | 0.077 | 0.015 | 0.067 | 0.012 | 0.012 | 0.002 | 0.130* | 0.037* |
|  |  | (0.044) | (0.010) | (0.093) | (0.014) | (0.048) | (0.009) | (0.067) | (0.012) | (0.058) | (0.009) | (0.051) | (0.015) |
|  | z-value | 0.670 | 0.400 |  |  | -0.12 | -0.2 |  |  | 1.530 | 2.070* |  |  |
|  | 2012 | 0.060** | 0.044** | 0.042 | 0.024 | 0.059* | 0.044* | 0.057* | 0.034* | 0.014 | 0.009 | 0.099** | 0.084** |
|  |  | (0.021) | (0.015) | (0.036) | (0.021) | (0.025) | (0.018) | (0.028) | (0.016) | (0.024) | (0.014) | (0.032) | (0.027) |
|  | z-value | -0.440 | -0.760 |  |  | -0.050 | -0.390 |  |  | 2.130* | 2.470* |  |  |
|  | 2010 | 0.148** | 0.028** | 0.040 | 0.007 | 0.118* | 0.026* | 0.112 | 0.017 | 0.095 | 0.015 | 0.070 | 0.020 |
|  |  | (0.054) | (0.010) | (0.070) | (0.012) | (0.057) | (0.012) | (0.067) | (0.010) | (0.057) | (0.009) | (0.065) | (0.018) |
|  | z-value | -1.22 | -1.3 |  |  | -0.07 | -0.57 |  |  | -0.29 | 0.26 |  |  |
| **2-week health status** | 2022 | 0.111**** | 0.095**** | 0.074* | 0.060* | 0.081** | 0.073** | 0.111** | 0.084** | 0.062* | 0.046* | 0.068* | 0.065* |
|  |  | (0.022) | (0.019) | (0.036) | (0.029) | (0.031) | (0.028) | (0.034) | (0.026) | (0.025) | (0.019) | (0.027) | (0.026) |
|  | z-value | -0.88 | -1.02 |  |  | 0.65 | 0.28 |  |  | 0.16 | 0.58 |  |  |
|  | 2020 | 0.081** | 0.065** | 0.052 | 0.044 | 0.054 | 0.046 | 0.073** | 0.056** | 0.010 | 0.008 | 0.076* | 0.072* |
|  |  | (0.028) | (0.022) | (0.033) | (0.027) | (0.032) | (0.028) | (0.026) | (0.020) | (0.028) | (0.021) | (0.034) | (0.032) |
|  | z-value | -0.66 | -0.62 |  |  | 0.47 | 0.29 |  |  | 1.5 | 1.68 |  |  |
|  | 2018 | 0.094**** | 0.087**** | 0.122**** | 0.111**** | 0.105**** | 0.100**** | 0.107**** | 0.092**** | 0.111**** | 0.094**** | 0.004 | 0.004 |
|  |  | (0.022) | (0.021) | (0.025) | (0.022) | (0.025) | (0.024) | (0.022) | (0.019) | (0.022) | (0.019) | (0.029) | (0.029) |
|  | z-value | 0.83 | 0.81 |  |  | 0.09 | -0.27 |  |  | -2.910** | -2.590** |  |  |
|  | 2016 | 0.083* | 0.074* | 0.056* | 0.051* | 0.088** | 0.083** | 0.048 | 0.040 | 0.021 | 0.017 | 0.054 | 0.054 |
|  |  | (0.034) | (0.030) | (0.026) | (0.024) | (0.028) | (0.027) | (0.032) | (0.026) | (0.029) | (0.024) | (0.031) | (0.030) |
|  | z-value | -0.62 | -0.59 |  |  | -0.94 | -1.16 |  |  | 0.78 | 0.96 |  |  |
|  | 2014 | 0.009 | 0.008 | -0.039 | -0.033 | -0.037 | -0.034 | 0.021 | 0.016 | -0.050* | -0.040* | 0.024 | 0.023 |
|  |  | (0.024) | (0.021) | (0.031) | (0.026) | (0.025) | (0.023) | (0.025) | (0.020) | (0.020) | (0.016) | (0.031) | (0.031) |
|  | z-value | -1.230 | -1.220 |  |  | 1.630 | 1.650 |  |  | 2.000* | 1.830 |  |  |
|  | 2012 | 0.003 | 0.002 | 0.012 | 0.009 | 0.001 | 0.001 | 0.017 | 0.013 | -0.023 | -0.017 | -0.002 | -0.002 |
|  |  | (0.034) | (0.030) | (0.034) | (0.027) | (0.036) | (0.032) | (0.029) | (0.022) | (0.030) | (0.023) | (0.035) | (0.035) |
|  | z-value | 0.190 | 0.180 |  |  | 0.340 | 0.300 |  |  | 0.450 | 0.370 |  |  |
|  | 2010 | 0.053 | 0.042 | 0.076* | 0.058* | 0.061* | 0.051* | 0.064* | 0.044* | 0.053* | -0.010 | 0.038* | -0.009 |
|  |  | (0.031) | (0.024) | (0.035) | (0.026) | (0.026) | (0.022) | (0.028) | (0.019) | (0.026) | (0.033) | (0.019) | (0.030) |
|  | z-value | 0.49 | 0.45 |  |  | 0.08 | -0.23 |  |  | -1.49 | -1.31 |  |  |

**Table S2** (Continued).

| **Health variables** | **Year** | **Rural** | | **Urban** | | **Female** | | **Male** | | **Age<60** | | **Age≥60** | |
| --- | --- | --- | --- | --- | --- | --- | --- | --- | --- | --- | --- | --- | --- |
|  |  | **WI** | **EI** | **WI** | **EI** | **WI** | **EI** | **WI** | **EI** | **WI** | **EI** | **WI** | **EI** |
| **Chronic disease status** | 2022 | 0.099* | 0.059* | 0.084 | 0.047 | 0.101* | 0.061* | 0.086* | 0.048* | 0.100 | 0.042 | -0.019 | -0.016 |
|  |  | (0.040) | (0.024) | (0.050) | (0.028) | (0.044) | (0.027) | (0.040) | (0.022) | (0.057) | (0.024) | (0.032) | (0.027) |
|  | z-value | -0.24 | -0.34 |  |  | -0.25 | -0.4 |  |  | -1.83 | -1.63 |  |  |
|  | 2020 | 0.050 | 0.027 | 0.119** | 0.054** | 0.057 | 0.031 | 0.106* | 0.051* | -0.008 | -0.003 | 0.055 | 0.043 |
|  |  | (0.041) | (0.022) | (0.045) | (0.020) | (0.031) | (0.017) | (0.045) | (0.022) | (0.040) | (0.015) | (0.036) | (0.028) |
|  | z-value | 1.14 | 0.88 |  |  | 0.89 | 0.75 |  |  | 1.17 | 1.44 |  |  |
|  | 2018 | 0.087** | 0.051** | 0.011 | 0.006 | 0.043 | 0.026 | 0.097** | 0.049** | 0.039 | 0.015 | -0.019 | -0.015 |
|  |  | (0.032) | (0.019) | (0.039) | (0.019) | (0.032) | (0.019) | (0.034) | (0.017) | (0.035) | (0.014) | (0.035) | (0.029) |
|  | z-value | -1.5 | -1.72 |  |  | 1.15 | 0.91 |  |  | -1.17 | -0.97 |  |  |
|  | 2016 | 0.067 | 0.037 | 0.065 | 0.043 | 0.043 | 0.028 | 0.078* | 0.044* | 0.007 | 0.003 | 0.002 | 0.002 |
|  |  | (0.037) | (0.020) | (0.039) | (0.026) | (0.035) | (0.023) | (0.036) | (0.021) | (0.034) | (0.015) | (0.039) | (0.033) |
|  | z-value | -0.02 | 0.19 |  |  | 0.7 | 0.53 |  |  | -0.09 | -0.03 |  |  |
|  | 2014 | -0.030 | -0.016 | 0.013 | 0.006 | -0.016 | -0.009 | -0.001 | <-0.001 | -0.030 | -0.012 | -0.060 | -0.049 |
|  |  | (0.027) | (0.014) | (0.035) | (0.017) | (0.028) | (0.016) | (0.031) | (0.014) | (0.025) | (0.010) | (0.033) | (0.027) |
|  | z-value | 0.97 | 1.000 |  |  | 0.370 | 0.420 |  |  | -0.720 | -1.290 |  |  |
|  | 2012 | -0.019 | -0.008 | -0.007 | -0.003 | -0.014 | -0.006 | -0.009 | -0.003 | -0.050 | -0.015 | -0.045 | -0.028 |
|  |  | (0.036) | (0.015) | (0.041) | (0.015) | (0.033) | (0.014) | (0.039) | (0.014) | (0.031) | (0.010) | (0.043) | (0.027) |
|  | z-value | 0.230 | 0.260 |  |  | 0.100 | 0.140 |  |  | 0.100 | -0.450 |  |  |
|  | 2010 | 0.050 | 0.024 | 0.045 | 0.020 | 0.049 | 0.024 | 0.053 | 0.023 | 0.003 | 0.001 | 0.038 | 0.027 |
|  |  | (0.033) | (0.015) | (0.041) | (0.018) | (0.031) | (0.015) | (0.038) | (0.016) | (0.027) | (0.010) | (0.045) | (0.032) |
|  | z-value | -0.1 | -0.17 |  |  | 0.09 | -0.06 |  |  | 0.66 | 0.77 |  |  |

* p < 0.05, ** p < 0.01, *** p < 0.001, **** p < 0.0001

**Table S3** RIF-Oaxaca decomposition of rural-urban health inequalities in China, 2016-2022

| **Variable** | **Self-rated health status** | | | | **Mental health status** | | | | **2-week health status** | | | | **Chronic disease status** | | | |
| --- | --- | --- | --- | --- | --- | --- | --- | --- | --- | --- | --- | --- | --- | --- | --- | --- |
|  | **EI** | | **WI** | | **EI** | | **WI** | | **EI** | | **WI** | | **EI** | | **WI** | |
| **Rural** | 0.077*** | (0.006) | 0.078*** | (0.006) | 0.055*** | (0.008) | 0.069*** | (0.010) | 0.102*** | (0.008) | 0.117*** | (0.009) | 0.040*** | (0.007) | 0.069*** | (0.012) |
| **Counterfactual group** | 0.083*** | (0.016) | 0.084*** | (0.016) | 0.064*** | (0.021) | 0.085*** | (0.027) | 0.053** | (0.022) | 0.058** | (0.025) | 0.032* | (0.019) | 0.053* | (0.031) |
| **Urban** | 0.072*** | (0.013) | 0.071*** | (0.013) | 0.054*** | (0.018) | 0.073*** | (0.024) | 0.057*** | (0.019) | 0.066*** | (0.022) | 0.039** | (0.015) | 0.072** | (0.028) |
| Tdifference | 0.005 | (0.014) | 0.007 | (0.014) | 0.002 | (0.020) | -0.004 | (0.026) | 0.044** | (0.020) | 0.050** | (0.023) | 0.001 | (0.017) | -0.003 | (0.030) |
| ToT_  Explained | 0.011 | (0.012) | 0.014 | (0.012) | 0.010 | (0.015) | 0.012 | (0.019) | -0.005 | (0.016) | -0.008 | (0.018) | -0.007 | (0.014) | -0.018 | (0.022) |
| ToT_  Unexplained | -0.006 | (0.022) | -0.007 | (0.022) | -0.008 | (0.029) | -0.016 | (0.038) | 0.049 | (0.031) | 0.058* | (0.034) | 0.007 | (0.026) | 0.016 | (0.043) |
|  | Explained | Unexplained | Explained | Unexplained | Explained | Unexplained | Explained | Unexplained | Explained | Unexplained | Explained | Unexplained | Explained | Unexplained | Explained | Unexplained |
| **Total** | 0.011 | -0.006 | 0.014 | -0.007 | 0.010 | -0.008 | 0.012 | -0.016 | -0.005 | 0.049 | -0.008 | 0.058* | -0.007 | 0.007 | -0.018 | 0.016 |
| Pure_explaine-d | 0.018** |  | 0.019*** |  | 0.024** |  | 0.029** |  | 0.015 |  | 0.014 |  | 0.005 |  | 0.006 |  |
|  | (0.007) |  | (0.007) |  | (0.010) |  | (0.013) |  | (0.010) |  | (0.012) |  | (0.008) |  | (0.015) |  |
| Pure_Unexpla-ined |  | -0.001 |  | -0.002 |  | -0.009 |  | -0.016 |  | 0.054* |  | 0.063* |  | 0.008 |  | 0.015 |
|  |  | (0.023) |  | (0.023) |  | (0.031) |  | (0.040) |  | (0.032) |  | (0.035) |  | (0.026) |  | (0.042) |
| Specif_err | -0.007 |  | -0.005 |  | -0.014 |  | -0.017 |  | -0.019** |  | -0.021** |  | -0.012 |  | -0.024** |  |
|  | (0.007) |  | (0.007) |  | (0.009) |  | (0.011) |  | (0.009) |  | (0.010) |  | (0.008) |  | (0.012) |  |
| Reweight_err |  | -0.005 |  | -0.005 |  | <0.001 |  | <0.001 |  | -0.005 |  | -0.005 |  | -0.001 |  | <0.001 |
|  |  | (0.004) |  | (0.004) |  | (0.006) |  | (0.007) |  | (0.006) |  | (0.007) |  | (0.005) |  | (0.008) |
|  | Pure_  explained | Pure_  Unexplained | Pure_  explained | Pure_  Unexplained | Pure_  explained | Pure_  Unexplained | Pure_  explained | Pure_  Unexplained | Pure_  explained | Pure_  Unexplained | Pure_  explained | Pure_  Unexplained | Pure_  explained | Pure_  Unexplained | Pure_  explained | Pure_  Unexplained |
| **LnPI** | <0.001 | 0.023 | <0.001 | 0.022 | <0.001 | -0.024 | <0.001 | -0.029 | 0.001 | -0.010 | 0.001 | -0.004 | <0.001 | 0.010 | <0.001 | 0.021 |
|  | (<0.001) | (0.035) | (<0.001) | (0.035) | (<0.001) | (0.047) | (<0.001) | (0.061) | (<0.001) | (0.049) | (0.001) | (0.054) | (<0.001) | (0.043) | (<0.001) | (0.070) |
| **LnPCHI** | <0.001 | -0.587 | <0.001 | -0.591 | 0.002 | -0.616 | 0.003 | -0.749 | -0.003** | -0.892** | -0.004** | -0.970** | <-0.001 | -0.407 | <-0.001 | -0.683 |
|  | (0.001) | (0.360) | (0.001) | (0.362) | (0.002) | (0.493) | (0.002) | (0.627) | (0.002) | (0.399) | (0.002) | (0.434) | (0.002) | (0.344) | (0.004) | (0.560) |
| **LnMIRR** | 0.001* | -0.010 | 0.001* | -0.010 | <-0.001 | 0.020 | -0.001 | 0.023 | 0.001 | -0.015 | <0.001 | -0.012 | 0.001 | 0.002 | 0.001 | 0.016 |
|  | (<0.001) | (0.046) | (<0.001) | (0.046) | (0.001) | (0.069) | (0.001) | (0.089) | (0.001) | (0.074) | (0.001) | (0.082) | (0.001) | (0.069) | (0.001) | (0.114) |
| **Age** | 0.004* | <0.001 | 0.004* | <0.001 | 0.006** | <0.001 | 0.008** | <0.001 | 0.003 | 0.001 | 0.002 | 0.001 | 0.001 | 0.001 | -0.001 | 0.001 |
|  | (0.002) | (0.001) | (0.002) | (0.001) | (0.003) | (0.002) | (0.004) | (0.003) | (0.003) | (0.002) | (0.003) | (0.002) | (0.002) | (0.002) | (0.004) | (0.003) |
| **Age^2^** | 0.001 | -0.020 | 0.001 | -0.021 | 0.001 | -0.022 | 0.001 | -0.024 | 0.001 | -0.028 | 0.001 | -0.029 | 0.001 | -0.004 | 0.001 | -0.007 |
|  | (0.001) | (0.027) | (0.001) | (0.027) | (0.001) | (0.038) | (0.001) | (0.049) | (0.001) | (0.040) | (0.001) | (0.044) | (0.001) | (0.038) | (0.001) | (0.063) |

**Table S3** (Continued).

| **Variable** | **Self-rated health status** | | | | **Mental health status** | | | | **2-week health status** | | | | **Chronic disease status** | | | |
| --- | --- | --- | --- | --- | --- | --- | --- | --- | --- | --- | --- | --- | --- | --- | --- | --- |
|  | **EI** | | **WI** | | **EI** | | **WI** | | **EI** | | **WI** | | **EI** | | **WI** | |
|  | Pure_  explained | Pure_  Unexplained | Pure_  explained | Pure_  Unexplained | Pure_  explained | Pure_  Unexplained | Pure_  explained | Pure_  Unexplained | Pure_  explained | Pure_  Unexplained | Pure_  explained | Pure_  Unexplained | Pure_  explained | Pure_  Unexplained | Pure_  explained | Pure_  Unexplained |
| **Gender (Reference group: Female)** | | | | | | | | | | | | | | | | |
| Male | <-0.001 | 0.002 | <-0.001 | 0.002 | <0.001 | -0.010 | <0.001 | -0.015 | <0.001 | -0.015 | <0.001 | -0.016 | <0.001 | -0.032 | 0.001 | -0.052 |
|  | (<0.001) | (0.029) | (<0.001) | (0.029) | (<0.001) | (0.038) | (0.001) | (0.048) | (<0.001) | (0.040) | (<0.001) | (0.044) | (<0.001) | (0.035) | (0.001) | (0.057) |
| **Ethnicity (Reference group: Other ethnicities)** | | | | | | | | | | | | | | | | |
| Han Chinese | -0.004 | -0.031 | -0.004 | -0.028 | 0.005* | 0.065 | 0.007 | 0.081 | -0.003 | -0.031 | -0.004 | -0.033 | 0.002 | 0.067 | 0.004 | 0.112 |
|  | (0.002) | (0.057) | (0.002) | (0.057) | (0.003) | (0.068) | (0.004) | (0.088) | (0.003) | (0.076) | (0.004) | (0.086) | (0.002) | (0.053) | (0.004) | (0.087) |
| **Marital status (Reference group: Married/Cohabiting)** | | | | | | | | | | | | | | | | |
| Single | <0.001 | 0.014 | <0.001 | 0.014 | <0.001 | 0.005 | <0.001 | 0.005 | 0.001 | 0.020 | 0.001 | 0.022 | 0.001 | 0.013 | 0.001 | 0.022 |
|  | (<0.001) | (0.015) | (<0.001) | (0.015) | (0.001) | (0.019) | (0.001) | (0.024) | (0.001) | (0.021) | (0.001) | (0.023) | (0.001) | (0.018) | (0.001) | (0.030) |
| Divorced/  Widowed | <0.001 | 0.004 | <0.001 | 0.004 | 0.001 | 0.003 | 0.002 | 0.004 | -0.001 | -0.009 | -0.001 | -0.009 | 0.001* | 0.001 | 0.003* | 0.002 |
|  | (0.001) | (0.007) | (0.001) | (0.007) | (0.001) | (0.011) | (0.001) | (0.014) | (0.001) | (0.011) | (0.001) | (0.012) | (0.001) | (0.010) | (0.001) | (0.016) |
| **Years of education** | 0.007** | 0.028 | 0.007** | 0.028 | 0.010** | 0.076 | 0.013** | 0.101 | 0.001 | 0.020 | 0.001 | 0.026 | 0.002 | 0.001 | 0.003 | 0.010 |
|  | (0.003) | (0.038) | (0.003) | (0.039) | (0.004) | (0.048) | (0.006) | (0.062) | (0.004) | (0.051) | (0.005) | (0.056) | (0.004) | (0.046) | (0.008) | (0.076) |
| **Agricultural occupation (Reference group: Non-agricultural)** | | | | | | | | | | | | | | | | |
| Agricultural | -0.002 | -0.022 | -0.002 | -0.021 | -0.009 | 0.012 | -0.013 | 0.017 | -0.007 | -0.021 | -0.009 | -0.026 | -0.013** | -0.014 | -0.023** | -0.023 |
|  | (0.005) | (0.026) | (0.005) | (0.026) | (0.007) | (0.036) | (0.009) | (0.047) | (0.007) | (0.037) | (0.008) | (0.041) | (0.006) | (0.031) | (0.011) | (0.051) |
| **Self-rated social status (Reference group: Average)** | | | | | | | | | | | | | | | | |
| Relatively low | -0.001 | -0.004 | -0.001 | -0.004 | -0.007*** | -0.026 | -0.009*** | -0.033 | -0.003 | -0.033 | -0.003 | -0.036 | -0.001 | -0.004 | -0.001 | -0.008 |
|  | (0.001) | (0.014) | (0.001) | (0.014) | (0.002) | (0.020) | (0.002) | (0.025) | (0.002) | (0.020) | (0.002) | (0.023) | (0.001) | (0.017) | (0.002) | (0.027) |
| Relatively high | <0.001 | 0.005 | <0.001 | 0.004 | 0.003 | -0.001 | 0.004 | <-0.001 | 0.001 | -0.007 | 0.002 | -0.008 | -0.001 | 0.021 | -0.002 | 0.036 |
|  | (0.002) | (0.016) | (0.002) | (0.017) | (0.002) | (0.022) | (0.003) | (0.029) | (0.002) | (0.024) | (0.003) | (0.026) | (0.002) | (0.021) | (0.004) | (0.035) |
| **Past-month smoking (Reference group: No)** | | | | | | | | | | | | | | | | |
| Yes | <0.001 | -0.016 | <0.001 | -0.017 | <-0.001 | 0.011 | <-0.001 | 0.015 | <-0.001 | 0.004 | <-0.001 | 0.005 | <-0.001 | 0.014 | <-0.001 | 0.025 |
|  | (<0.001) | (0.018) | (<0.001) | (0.018) | (<0.001) | (0.024) | (<0.001) | (0.031) | (<0.001) | (0.025) | (<0.001) | (0.028) | (<0.001) | (0.022) | (0.001) | (0.036) |
| **Frequent past-month alcohol use (Reference group: No)** | | | | | | | | | | | | | | | | |
| Yes | -0.001* | 0.013 | -0.001* | 0.013 | <-0.001 | -0.010 | <-0.001 | -0.013 | <-0.001 | -0.007 | <-0.001 | -0.009 | <-0.001 | -0.002 | -0.001 | -0.004 |
|  | (<0.001) | (0.010) | (<0.001) | (0.010) | (<0.001) | (0.013) | (0.001) | (0.017) | (0.001) | (0.014) | (0.001) | (0.016) | (<0.001) | (0.012) | (0.001) | (0.020) |
| **Nap habit (Reference group: No)** | | | | | | | | | | | | | | | | |
| Yes | <-0.001 | 0.005 | <-0.001 | 0.005 | <-0.001 | 0.022 | <-0.001 | 0.026 | <0.001 | -0.006 | <0.001 | -0.005 | <-0.001 | 0.029 | <-0.001 | 0.049 |
|  | (<0.001) | (0.023) | (<0.001) | (0.023) | (<0.001) | (0.032) | (0.001) | (0.041) | (<0.001) | (0.033) | (0.001) | (0.036) | (<0.001) | (0.027) | (0.001) | (0.044) |

**Table S3** (Continued).

| **Variable** | **Self-rated health status** | | | | **Mental health status** | | | | **2-week health status** | | | | **Chronic disease status** | | | |
| --- | --- | --- | --- | --- | --- | --- | --- | --- | --- | --- | --- | --- | --- | --- | --- | --- |
|  | **EI** | | **WI** | | **EI** | | **WI** | | **EI** | | **WI** | | **EI** | | **WI** | |
|  | Pure_  explained | Pure_  Unexplained | Pure_  explained | Pure_  Unexplained | Pure_  explained | Pure_  Unexplained | Pure_  explained | Pure_  Unexplained | Pure_  explained | Pure_  Unexplained | Pure_  explained | Pure_  Unexplained | Pure_  explained | Pure_  Unexplained | Pure_  explained | Pure_  Unexplained |
| **Weekly exercise (Reference group: No)** | | | | | | | | | | | | | | | | |
| Yes | <-0.001 | 0.008 | <0.001 | 0.007 | -0.002 | 0.006 | -0.004 | 0.005 | <0.001 | <-0.001 | <0.001 | 0.001 | <-0.001 | -0.001 | <-0.001 | -0.004 |
|  | (0.001) | (0.014) | (0.001) | (0.014) | (0.002) | (0.019) | (0.002) | (0.025) | (0.002) | (0.021) | (0.002) | (0.023) | (0.002) | (0.017) | (0.003) | (0.028) |
| **Life satisfaction (Reference group: Somewhat satisfied)** | | | | | | | | | | | | | | | | |
| Dissatisfied/  Neutral | -0.001 | 0.013 | -0.001 | 0.012 | <0.001 | 0.020 | 0.002 | 0.029 | <-0.001 | -0.003 | <-0.001 | -0.003 | 0.002** | 0.027 | 0.005** | 0.041 |
|  | (0.001) | (0.019) | (0.001) | (0.019) | (0.001) | (0.027) | (0.002) | (0.035) | (0.001) | (0.028) | (0.001) | (0.031) | (0.001) | (0.024) | (0.002) | (0.039) |
| Very satisfied | <-0.001 | 0.031 | <-0.001 | 0.030 | -0.001 | 0.014 | <-0.001 | 0.019 | -0.001 | 0.029 | -0.001 | 0.034 | -0.004*** | 0.031 | -0.006*** | 0.049 |
|  | (0.001) | (0.020) | (0.001) | (0.020) | (0.001) | (0.026) | (0.001) | (0.034) | (0.001) | (0.028) | (0.001) | (0.031) | (0.001) | (0.024) | (0.002) | (0.040) |
| **Cooking water source (Reference group: Non-tap water)** | | | | | | | | | | | | | | | | |
| Tap water | <0.001 | 0.012 | 0.001 | 0.012 | <-0.001 | -0.064 | -0.001 | -0.082 | -0.003 | -0.024 | -0.004 | -0.028 | 0.003 | 0.009 | 0.005 | 0.017 |
|  | (0.003) | (0.031) | (0.003) | (0.031) | (0.004) | (0.042) | (0.006) | (0.055) | (0.004) | (0.044) | (0.005) | (0.049) | (0.004) | (0.037) | (0.007) | (0.061) |
| **Cooking fuel type (Reference group: Non-clean fuels)** | | | | | | | | | | | | | | | | |
| Clean fuels | 0.010** | 0.006 | 0.010** | 0.006 | 0.015** | -0.003 | 0.019** | -0.003 | 0.024*** | 0.038 | 0.027*** | 0.041 | 0.008 | -0.007 | 0.013 | -0.016 |
|  | (0.005) | (0.023) | (0.005) | (0.023) | (0.007) | (0.031) | (0.009) | (0.040) | (0.007) | (0.032) | (0.008) | (0.036) | (0.006) | (0.027) | (0.011) | (0.045) |
| **Household size** | <0.001 | -0.034 | <0.001 | -0.033 | <0.001 | 0.010 | <0.001 | 0.017 | <0.001 | -0.012 | <0.001 | -0.019 | <0.001 | -0.007 | <0.001 | -0.014 |
|  | (<0.001) | (0.049) | (<0.001) | (0.049) | (<0.001) | (0.065) | (<0.001) | (0.084) | (<0.001) | (0.067) | (<0.001) | (0.075) | (<0.001) | (0.056) | (<0.001) | (0.092) |
| **Basic social medical insurance enrollment (Reference group: Not enrolled)** | | | | | | | | | | | | | | | | |
| Enrolled | <0.001 | 0.051 | <0.001 | 0.056 | -0.002 | 0.085 | -0.002 | 0.113 | -0.001 | 0.028 | -0.001 | 0.034 | 0.001 | 0.028 | 0.002 | 0.048 |
|  | (0.001) | (0.075) | (0.001) | (0.075) | (0.001) | (0.107) | (0.001) | (0.138) | (0.001) | (0.106) | (0.001) | (0.117) | (0.001) | (0.076) | (0.001) | (0.126) |
| **Level of medical expertise of the visited institution (Reference group: Fair)** | | | | | | | | | | | | | | | | |
| Low | <0.001 | -0.001 | <0.001 | -0.001 | <-0.001 | 0.012 | -0.001 | 0.015 | <-0.001 | 0.013 | <-0.001 | 0.014 | 0.001 | -0.004 | 0.001 | -0.006 |
|  | (<0.001) | (0.009) | (<0.001) | (0.009) | (<0.001) | (0.012) | (0.001) | (0.015) | (<0.001) | (0.013) | (<0.001) | (0.014) | (<0.001) | (0.011) | (0.001) | (0.018) |
| High | 0.003*** | -0.013 | 0.003*** | -0.013 | 0.001 | 0.004 | 0.001 | 0.008 | 0.003*** | -0.019 | 0.004*** | -0.023 | 0.001 | -0.005 | 0.002 | -0.008 |
|  | (0.001) | (0.024) | (0.001) | (0.024) | (0.001) | (0.032) | (0.001) | (0.042) | (0.001) | (0.035) | (0.001) | (0.039) | (0.001) | (0.028) | (0.002) | (0.046) |
| Year FE | Yes | Yes | Yes | Yes | Yes | Yes | Yes | Yes | Yes | Yes | Yes | Yes | Yes | Yes | Yes | Yes |

*, **, and *** denote significance at the 10%, 5%, and 1% levels, respectively. *PI* Personal Income. *PCHI* Per Capita Household Income. *MIRR* Medical Insurance Reimbursement Ratio.

**Table S4** RIF-Oaxaca decomposition of male-female health inequalities in China, 2016-2022

| **Variable** | **Self-rated health status** | | | | **Mental health status** | | | | **2-week health status** | | | | **Chronic disease status** | | | |
| --- | --- | --- | --- | --- | --- | --- | --- | --- | --- | --- | --- | --- | --- | --- | --- | --- |
|  | **EI** | | **WI** | | **EI** | | **WI** | | **EI** | | **WI** | | **EI** | | **WI** | |
| **Female** | 0.066*** | (0.008) | 0.066*** | (0.008) | 0.051*** | (0.011) | 0.061*** | (0.013) | 0.091*** | (0.012) | 0.098*** | (0.012) | 0.029*** | (0.010) | 0.047*** | (0.016) |
| **Counterfactual group** | 0.068*** | (0.025) | 0.070*** | (0.025) | 0.054** | (0.027) | 0.060 | (0.038) | 0.070** | (0.029) | 0.080** | (0.035) | 0.112*** | (0.032) | 0.184*** | (0.050) |
| **Male** | 0.079*** | (0.008) | 0.078*** | (0.008) | 0.065*** | (0.011) | 0.095*** | (0.016) | 0.074*** | (0.012) | 0.092*** | (0.014) | 0.047*** | (0.009) | 0.090*** | (0.018) |
| **Tdifference** | -0.012 | (0.011) | -0.012 | (0.011) | -0.013 | (0.016) | -0.033 | (0.021) | 0.017 | (0.016) | 0.006 | (0.019) | -0.018 | (0.013) | -0.043* | (0.024) |
| **ToT_**  **Explained** | -0.010 | (0.022) | -0.008 | (0.022) | -0.011 | (0.023) | -0.035 | (0.033) | -0.004 | (0.026) | -0.013 | (0.031) | 0.065** | (0.029) | 0.094** | (0.045) |
| **ToT_**  **Unexplained** | -0.002 | (0.026) | -0.004 | (0.026) | -0.002 | (0.029) | 0.002 | (0.041) | 0.021 | (0.032) | 0.018 | (0.038) | -0.083** | (0.034) | -0.137*** | (0.053) |
|  | Explained | Unexplained | Explained | Unexplained | Explained | Unexplained | Explained | Unexplained | Explained | Unexplained | Explained | Unexplained | Explained | Unexplained | Explained | Unexplained |
| **Total** | -0.010 | -0.002 | -0.008 | -0.004 | -0.011 | -0.002 | -0.035 | 0.002 | -0.004 | 0.021 | -0.013 | 0.018 | 0.065** | -0.083** | 0.094** | -0.137*** |
| Pure_  explained | -0.005 |  | -0.006 |  | 0.001 |  | -0.003 |  | -0.009 |  | -0.016 |  | 0.004 |  | -0.006 |  |
|  | (0.011) |  | (0.011) |  | (0.016) |  | (0.022) |  | (0.015) |  | (0.019) |  | (0.013) |  | (0.025) |  |
| Pure_  Unexplained |  | 0.005 |  | 0.003 |  | -0.010 |  | -0.010 |  | 0.022 |  | 0.018 |  | -0.081*** |  | -0.136*** |
|  |  | (0.022) |  | (0.022) |  | (0.026) |  | (0.036) |  | (0.028) |  | (0.033) |  | (0.025) |  | (0.040) |
| Specif_err | -0.005 |  | -0.001 |  | -0.012 |  | -0.032 |  | 0.004 |  | 0.003 |  | 0.061*** |  | 0.100*** |  |
|  | (0.016) |  | (0.016) |  | (0.016) |  | (0.022) |  | (0.018) |  | (0.022) |  | (0.023) |  | (0.034) |  |
| Reweight_err |  | -0.007 |  | -0.007 |  | 0.007 |  | 0.012 |  | <-0.001 |  | <0.001 |  | -0.002 |  | -0.001 |
|  |  | (0.008) |  | (0.008) |  | (0.012) |  | (0.018) |  | (0.010) |  | (0.011) |  | (0.013) |  | (0.020) |
|  | Pure_  explained | Pure_  Unexplained | Pure_  explained | Pure_  Unexplained | Pure_  explained | Pure_  Unexplained | Pure_  explained | Pure_  Unexplained | Pure_  explained | Pure_  Unexplained | Pure_  explained | Pure_  Unexplained | Pure_  explained | Pure_  Unexplained | Pure_  explained | Pure_  Unexplained |
| **LnPI** | -0.002 | 0.022 | -0.003 | 0.021 | -0.004 | -0.034 | -0.007 | -0.069 | -0.002 | 0.029 | -0.004 | 0.033 | -0.003 | 0.088 | -0.006 | 0.137 |
|  | (0.003) | (0.043) | (0.003) | (0.043) | (0.004) | (0.043) | (0.006) | (0.061) | (0.004) | (0.049) | (0.005) | (0.057) | (0.003) | (0.056) | (0.006) | (0.087) |
| **LnPCHI** | 0.003** | 0.195 | 0.003** | 0.177 | 0.008** | 0.884* | 0.011** | 1.240** | -0.001 | 0.100 | -0.001 | 0.112 | <-0.001 | -0.640** | <-0.001 | -1.065** |
|  | (0.001) | (0.263) | (0.002) | (0.263) | (0.003) | (0.479) | (0.005) | (0.614) | (0.001) | (0.433) | (0.002) | (0.494) | (0.001) | (0.308) | (0.002) | (0.496) |
| **LnMIRR** | 0.001 | 0.014 | 0.001 | 0.010 | <-0.001 | 0.037 | -0.001 | 0.043 | 0.002 | 0.086 | 0.001 | 0.090 | 0.001 | -0.084 | <-0.001 | -0.137 |
|  | (0.001) | (0.050) | (0.001) | (0.050) | (0.001) | (0.064) | (0.001) | (0.084) | (0.001) | (0.085) | (0.001) | (0.099) | (0.001) | (0.073) | (0.002) | (0.117) |
| **Age** | -0.001* | -0.001 | -0.001* | -0.002 | -0.003** | <-0.001 | -0.003* | <-0.001 | -0.002* | 0.001 | -0.002 | 0.001 | -0.001 | -0.001 | -0.001 | -0.002 |
|  | (0.001) | (0.001) | (0.001) | (0.001) | (0.001) | (0.001) | (0.002) | (0.001) | (0.001) | (0.001) | (0.001) | (0.001) | (0.001) | (0.001) | (0.002) | (0.002) |
| **Age^2^** | <0.001 | -0.026 | <0.001 | -0.025 | <0.001 | -0.004 | <0.001 | -0.014 | <0.001 | 0.024 | <0.001 | 0.028 | <0.001 | 0.019 | <0.001 | 0.037 |
|  | (0.001) | (0.029) | (0.001) | (0.029) | (<0.001) | (0.033) | (<0.001) | (0.042) | (0.001) | (0.038) | (0.001) | (0.044) | (0.001) | (0.040) | (0.002) | (0.063) |

**Table S4** (Continued).

| **Variable** | **Self-rated health status** | | | | **Mental health status** | | | | **2-week health status** | | | | **Chronic disease status** | | | |
| --- | --- | --- | --- | --- | --- | --- | --- | --- | --- | --- | --- | --- | --- | --- | --- | --- |
|  | **EI** | | **WI** | | **EI** | | **WI** | | **EI** | | **WI** | | **EI** | | **WI** | |
|  | Pure_  explained | Pure_  Unexplained | Pure_  explained | Pure_  Unexplained | Pure_  explained | Pure_  Unexplained | Pure_  explained | Pure_  Unexplained | Pure_  explained | Pure_  Unexplained | Pure_  explained | Pure_  Unexplained | Pure_  explained | Pure_  Unexplained | Pure_  explained | Pure_  Unexplained |
| **Residence (Reference group: Rural)** | | | | | | | | | | | | | | | | |
| Urban | <-0.001 | -0.016 | <-0.001 | -0.016 | <0.001 | -0.006 | <0.001 | -0.012 | -0.001 | -0.012 | -0.001 | -0.012 | <-0.001 | -0.051* | <-0.001 | -0.078 |
|  | (<0.001) | (0.022) | (<0.001) | (0.022) | (<0.001) | (0.024) | (0.001) | (0.031) | (0.001) | (0.028) | (0.001) | (0.033) | (<0.001) | (0.030) | (0.001) | (0.048) |
| **Ethnicity (Reference group: Other ethnicities)** | | | | | | | | | | | | | | | | |
| Han Chinese | <-0.001 | -0.027 | <-0.001 | -0.023 | <-0.001 | 0.028 | <-0.001 | 0.051 | <-0.001 | -0.022 | <-0.001 | -0.028 | <-0.001 | -0.115** | <-0.001 | -0.186** |
|  | (<0.001) | (0.048) | (<0.001) | (0.048) | (<0.001) | (0.060) | (<0.001) | (0.078) | (0.001) | (0.066) | (0.001) | (0.077) | (0.001) | (0.053) | (0.001) | (0.084) |
| **Marital status (Reference group: Married/Cohabiting)** | | | | | | | | | | | | | | | | |
| Single | 0.001 | 0.011 | 0.001 | 0.011 | <0.001 | -0.004 | 0.001 | <-0.001 | <0.001 | -0.036** | <0.001 | -0.042** | 0.001 | -0.005 | 0.002 | -0.010 |
|  | (0.001) | (0.011) | (0.001) | (0.011) | (0.001) | (0.014) | (0.002) | (0.018) | (0.001) | (0.015) | (0.001) | (0.018) | (0.001) | (0.010) | (0.002) | (0.017) |
| Divorced/  Widowed | -0.001 | -0.001 | -0.001 | -0.002 | -0.005 | 0.034** | -0.010 | 0.049** | 0.002 | -0.007 | 0.002 | -0.009 | -0.006 | -0.007 | -0.012 | -0.011 |
|  | (0.004) | (0.016) | (0.004) | (0.016) | (0.005) | (0.014) | (0.008) | (0.020) | (0.005) | (0.018) | (0.006) | (0.021) | (0.004) | (0.022) | (0.008) | (0.034) |
| **Years of education** | -0.002 | 0.014 | -0.003 | 0.013 | -0.003 | -0.028 | -0.007 | -0.044 | -0.008* | 0.007 | -0.012* | 0.002 | <-0.001 | 0.105** | -0.001 | 0.176** |
|  | (0.003) | (0.037) | (0.003) | (0.038) | (0.005) | (0.042) | (0.007) | (0.055) | (0.005) | (0.051) | (0.006) | (0.060) | (0.004) | (0.050) | (0.008) | (0.079) |
| **Agricultural occupation (Reference group: Non-agricultural)** | | | | | | | | | | | | | | | | |
| Agricultural | -0.001 | -0.003 | -0.001 | -0.005 | -0.001 | 0.067** | -0.001 | 0.089** | -0.001 | -0.032 | -0.001 | -0.036 | -0.001 | -0.032 | -0.002 | -0.050 |
|  | (0.001) | (0.029) | (0.001) | (0.029) | (0.001) | (0.032) | (0.001) | (0.042) | (0.001) | (0.035) | (0.001) | (0.040) | (0.001) | (0.039) | (0.002) | (0.061) |
| **Self-rated social status (Reference group: Average)** | | | | | | | | | | | | | | | | |
| Relatively low | <0.001 | -0.022 | <0.001 | -0.022 | 0.001 | -0.016 | 0.001 | -0.026 | <-0.001 | 0.037* | <-0.001 | 0.047** | <0.001 | -0.017 | <0.001 | -0.023 |
|  | (<0.001) | (0.017) | (<0.001) | (0.018) | (0.001) | (0.019) | (0.001) | (0.026) | (<0.001) | (0.020) | (<0.001) | (0.023) | (<0.001) | (0.021) | (0.001) | (0.033) |
| Relatively high | <0.001 | -0.016 | <0.001 | -0.017 | 0.001 | -0.017 | 0.001 | -0.023 | <0.001 | 0.009 | <0.001 | 0.013 | <0.001 | 0.001 | <0.001 | 0.003 |
|  | (<0.001) | (0.015) | (<0.001) | (0.015) | (0.001) | (0.019) | (0.001) | (0.026) | (<0.001) | (0.021) | (0.001) | (0.025) | (<0.001) | (0.016) | (0.001) | (0.026) |
| **Past-month smoking (Reference group: No)** | | | | | | | | | | | | | | | | |
| Yes | -0.008 | -0.002 | -0.009 | -0.002 | 0.006 | -0.002 | 0.012 | -0.003 | -0.007 | -0.001 | -0.010 | -0.001 | 0.005 | 0.002 | 0.002 | 0.003 |
|  | (0.008) | (0.002) | (0.008) | (0.002) | (0.011) | (0.003) | (0.016) | (0.003) | (0.011) | (0.003) | (0.014) | (0.003) | (0.009) | (0.002) | (0.017) | (0.004) |
| **Frequent past-month alcohol use (Reference group: No)** | | | | | | | | | | | | | | | | |
| Yes | 0.001 | -0.001 | 0.001 | -0.001 | 0.003 | 0.002 | 0.003 | 0.002 | 0.006 | 0.002 | 0.007 | 0.002 | 0.007 | 0.003 | 0.011 | 0.005 |
|  | (0.004) | (0.002) | (0.004) | (0.002) | (0.006) | (0.003) | (0.008) | (0.003) | (0.006) | (0.003) | (0.007) | (0.004) | (0.005) | (0.002) | (0.009) | (0.004) |
| **Nap habit (Reference group: No)** | | | | | | | | | | | | | | | | |
| Yes | <0.001 | -0.013 | <0.001 | -0.013 | <0.001 | 0.017 | <0.001 | 0.023 | 0.001 | -0.016 | 0.001 | -0.016 | 0.001 | -0.083** | 0.001 | -0.128** |
|  | (<0.001) | (0.029) | (<0.001) | (0.029) | (<0.001) | (0.030) | (0.001) | (0.040) | (0.001) | (0.036) | (0.001) | (0.042) | (0.001) | (0.039) | (0.001) | (0.061) |

**Table S4** (Continued).

| **Variable** | **Self-rated health status** | | | | **Mental health status** | | | | **2-week health status** | | | | **Chronic disease status** | | | |
| --- | --- | --- | --- | --- | --- | --- | --- | --- | --- | --- | --- | --- | --- | --- | --- | --- |
|  | **EI** | | **WI** | | **EI** | | **WI** | | **EI** | | **WI** | | **EI** | | **WI** | |
|  | Pure_  explained | Pure_  Unexplained | Pure_  explained | Pure_  Unexplained | Pure_  explained | Pure_  Unexplained | Pure_  explained | Pure_  Unexplained | Pure_  explained | Pure_  Unexplained | Pure_  explained | Pure_  Unexplained | Pure_  explained | Pure_  Unexplained | Pure_  explained | Pure_  Unexplained |
| **Weekly exercise (Reference group: No)** | | | | | | | | | | | | | | | | |
| Yes | <0.001 | 0.009 | <0.001 | 0.008 | <-0.001 | 0.010 | <-0.001 | 0.008 | 0.001 | 0.034* | 0.001 | 0.041* | 0.001 | 0.042** | 0.001 | 0.068** |
|  | (<0.001) | (0.013) | (<0.001) | (0.014) | (<0.001) | (0.018) | (0.001) | (0.025) | (0.001) | (0.019) | (0.001) | (0.022) | (<0.001) | (0.017) | (0.001) | (0.028) |
| **Life satisfaction (Reference group: Somewhat satisfied)** | | | | | | | | | | | | | | | | |
| Dissatisfied/  Neutral | <0.001 | 0.008 | <0.001 | 0.009 | <-0.001 | 0.041* | -0.001 | 0.060* | <-0.001 | 0.040 | <-0.001 | 0.043 | <-0.001 | -0.005 | <-0.001 | -0.006 |
|  | (<0.001) | (0.018) | (<0.001) | (0.018) | (0.001) | (0.024) | (0.002) | (0.033) | (0.001) | (0.024) | (0.001) | (0.028) | (<0.001) | (0.019) | (<0.001) | (0.031) |
| Very satisfied | <0.001 | -0.012 | <0.001 | -0.013 | -0.001 | 0.017 | -0.001 | 0.020 | -0.001 | -0.016 | -0.001 | -0.020 | -0.001 | <0.001 | -0.001 | 0.004 |
|  | (<0.001) | (0.016) | (<0.001) | (0.016) | (0.001) | (0.023) | (0.001) | (0.030) | (0.001) | (0.025) | (0.001) | (0.029) | (0.001) | (0.020) | (0.001) | (0.032) |
| **Cooking water source (Reference group: Non-tap water)** | | | | | | | | | | | | | | | | |
| Tap water | <-0.001 | 0.015 | <-0.001 | 0.014 | <-0.001 | 0.060 | <-0.001 | 0.089 | <-0.001 | 0.013 | <-0.001 | 0.012 | <-0.001 | 0.013 | <-0.001 | 0.026 |
|  | (<0.001) | (0.032) | (<0.001) | (0.032) | (<0.001) | (0.041) | (<0.001) | (0.056) | (<0.001) | (0.040) | (<0.001) | (0.047) | (<0.001) | (0.033) | (<0.001) | (0.054) |
| **Cooking fuel type (Reference group: Non-clean fuels)** | | | | | | | | | | | | | | | | |
| Clean fuels | <-0.001 | -0.013 | <-0.001 | -0.013 | <-0.001 | -0.058 | <-0.001 | -0.082 | <0.001 | -0.027 | <0.001 | -0.026 | <-0.001 | -0.002 | <-0.001 | <-0.001 |
|  | (<0.001) | (0.033) | (<0.001) | (0.034) | (<0.001) | (0.039) | (<0.001) | (0.053) | (<0.001) | (0.039) | (<0.001) | (0.046) | (<0.001) | (0.038) | (<0.001) | (0.061) |
| **Household size** | 0.002 | -0.028 | 0.001 | -0.026 | 0.001 | -0.002 | 0.001 | -0.007 | 0.001 | -0.103* | 0.001 | -0.112 | 0.001 | -0.070 | 0.002 | -0.108 |
|  | (0.001) | (0.044) | (0.001) | (0.044) | (0.001) | (0.056) | (0.001) | (0.074) | (0.001) | (0.061) | (0.001) | (0.071) | (0.001) | (0.049) | (0.001) | (0.079) |
| **Basic social medical insurance enrollment (Reference group: Not enrolled)** | | | | | | | | | | | | | | | | |
| Enrolled | <-0.001 | -0.123* | <-0.001 | -0.121* | <0.001 | -0.050 | <0.001 | -0.047 | <0.001 | 0.166 | <0.001 | 0.184 | -0.001 | -0.160* | -0.001 | -0.243* |
|  | (0.001) | (0.067) | (0.001) | (0.068) | (<0.001) | (0.093) | (0.001) | (0.119) | (<0.001) | (0.137) | (0.001) | (0.160) | (0.001) | (0.090) | (0.001) | (0.139) |
| **Level of medical expertise of the visited institution (Reference group: Fair)** | | | | | | | | | | | | | | | | |
| Low | -0.001 | -0.003 | -0.001 | -0.003 | -0.001 | -0.007 | <-0.001 | -0.011 | -0.001 | 0.007 | -0.001 | 0.010 | -0.003*** | -0.004 | -0.006*** | -0.008 |
|  | (0.001) | (0.006) | (0.001) | (0.006) | (0.001) | (0.008) | (0.002) | (0.011) | (0.001) | (0.008) | (0.002) | (0.009) | (0.001) | (0.006) | (0.002) | (0.010) |
| High | 0.004*** | -0.056 | 0.004*** | -0.057 | <0.001 | -0.015 | <0.001 | -0.034 | 0.003 | <-0.001 | 0.004 | 0.002 | 0.004** | -0.046 | 0.007** | -0.067 |
|  | (0.002) | (0.036) | (0.002) | (0.036) | (0.002) | (0.035) | (0.003) | (0.049) | (0.002) | (0.036) | (0.002) | (0.042) | (0.002) | (0.045) | (0.003) | (0.071) |
| Year FE | Yes | Yes | Yes | Yes | Yes | Yes | Yes | Yes | Yes | Yes | Yes | Yes | Yes | Yes | Yes | Yes |

*, **, and *** denote significance at the 10%, 5%, and 1% levels, respectively. *PI* Personal Income. *PCHI* Per Capita Household Income. *MIRR* Medical Insurance Reimbursement Ratio.

**Table S5** RIF-Oaxaca decomposition of age-related health inequalities in China, 2016-2022

| **Variable** | **Self-rated health status** | | | | **Mental health status** | | | | **2-week health status** | | | | **Chronic disease status** | | | |
| --- | --- | --- | --- | --- | --- | --- | --- | --- | --- | --- | --- | --- | --- | --- | --- | --- |
|  | **EI** | | **WI** | | **EI** | | **WI** | | **EI** | | **WI** | | **EI** | | **WI** | |
| **Age<60** | 0.024*** | (0.007) | 0.024*** | (0.007) | 0.020** | (0.010) | 0.028** | (0.013) | 0.037*** | (0.010) | 0.046*** | (0.013) | -0.003 | (0.007) | -0.007 | (0.018) |
| **Counterfactual group** | -0.023 | (0.022) | -0.023 | (0.023) | 0.092*** | (0.035) | 0.109*** | (0.042) | 0.025 | (0.040) | 0.026 | (0.043) | -0.038 | (0.040) | -0.050 | (0.053) |
| **Age≥60** | 0.073*** | (0.009) | 0.080*** | (0.010) | 0.101*** | (0.014) | 0.116*** | (0.016) | 0.078*** | (0.014) | 0.079*** | (0.014) | 0.014 | (0.013) | 0.017 | (0.016) |
| **Tdifference** | -0.049*** | (0.011) | -0.056*** | (0.012) | -0.081*** | (0.017) | -0.088*** | (0.021) | -0.041** | (0.017) | -0.033* | (0.019) | -0.017 | (0.015) | -0.023 | (0.024) |
| **ToT_**  **Explained** | -0.095*** | (0.021) | -0.104*** | (0.022) | -0.009 | (0.033) | -0.006 | (0.039) | -0.053 | (0.038) | -0.053 | (0.041) | -0.052 | (0.038) | -0.067 | (0.050) |
| **ToT_**  **Unexplained** | 0.047* | (0.024) | 0.047* | (0.026) | -0.072* | (0.038) | -0.081* | (0.046) | 0.012 | (0.044) | 0.020 | (0.047) | 0.035 | (0.044) | 0.043 | (0.059) |
|  | Explained | Unexplained | Explained | Unexplained | Explained | Unexplained | Explained | Unexplained | Explained | Unexplained | Explained | Unexplained | Explained | Unexplained | Explained | Unexplained |
| **Total** | -0.095*** | 0.047* | -0.104*** | 0.047* | -0.009 | -0.072* | -0.006 | -0.081* | -0.053 | 0.012 | -0.053 | 0.020 | -0.052 | 0.035 | -0.067 | 0.043 |
| Pure_  explained | -0.020** |  | -0.024** |  | 0.020 |  | 0.026 |  | 0.007 |  | 0.009 |  | -0.010 |  | -0.012 |  |
|  | (0.009) |  | (0.010) |  | (0.015) |  | (0.017) |  | (0.015) |  | (0.015) |  | (0.013) |  | (0.016) |  |
| Pure_  Unexplained |  | 0.054 |  | 0.055 |  | -0.012 |  | -0.009 |  | 0.055 |  | 0.067 |  | 0.071 |  | 0.089 |
|  |  | (0.034) |  | (0.036) |  | (0.058) |  | (0.069) |  | (0.071) |  | (0.076) |  | (0.075) |  | (0.099) |
| Specif_err | -0.075*** |  | -0.079*** |  | -0.029 |  | -0.033 |  | -0.060* |  | -0.062* |  | -0.041 |  | -0.054 |  |
|  | (0.018) |  | (0.018) |  | (0.028) |  | (0.033) |  | (0.033) |  | (0.035) |  | (0.033) |  | (0.044) |  |
| Reweight_err |  | -0.008 |  | -0.008 |  | -0.059 |  | -0.072 |  | -0.043 |  | -0.047 |  | -0.036 |  | -0.046 |
|  |  | (0.021) |  | (0.022) |  | (0.037) |  | (0.045) |  | (0.040) |  | (0.043) |  | (0.043) |  | (0.056) |
|  | Pure_  explained | Pure_  Unexplained | Pure_  explained | Pure_  Unexplained | Pure_  explained | Pure_  Unexplained | Pure_  explained | Pure_  Unexplained | Pure_  explained | Pure_  Unexplained | Pure_  explained | Pure_  Unexplained | Pure_  explained | Pure_  Unexplained | Pure_  explained | Pure_  Unexplained |
| **LnPI** | 0.002 | 0.011 | 0.001 | 0.015 | 0.008* | -0.020 | 0.010* | -0.030 | 0.004 | 0.046 | 0.004 | 0.054 | -0.019*** | 0.127* | -0.023*** | 0.189* |
|  | (0.003) | (0.038) | (0.003) | (0.040) | (0.005) | (0.067) | (0.005) | (0.080) | (0.005) | (0.073) | (0.005) | (0.078) | (0.004) | (0.072) | (0.005) | (0.097) |
| **LnPCHI** | -0.005*** | 0.311 | -0.006*** | 0.328 | -0.007** | 0.017 | -0.007** | -0.128 | 0.009*** | -0.134 | 0.009*** | -0.154 | <-0.001 | -0.054 | <-0.001 | -0.149 |
|  | (0.002) | (0.318) | (0.002) | (0.336) | (0.003) | (0.554) | (0.003) | (0.647) | (0.003) | (0.561) | (0.003) | (0.598) | (0.003) | (0.520) | (0.003) | (0.779) |
| **LnMIRR** | -0.001 | -0.029 | -0.003 | -0.021 | 0.003 | 0.047 | 0.005 | 0.051 | 0.004 | -0.041 | 0.005 | -0.039 | -0.001 | -0.015 | -0.002 | -0.012 |
|  | (0.002) | (0.052) | (0.002) | (0.054) | (0.003) | (0.075) | (0.003) | (0.091) | (0.003) | (0.078) | (0.003) | (0.085) | (0.003) | (0.084) | (0.004) | (0.118) |
| **Gender (Reference group: Female)** | | | | | | | | | | | | | | | | |
| Male | <-0.001 | -0.017 | <-0.001 | -0.017 | <-0.001 | -0.005 | <-0.001 | -0.008 | <-0.001 | -0.056 | <-0.001 | -0.059 | <-0.001 | -0.016 | <-0.001 | -0.016 |
|  | (<0.001) | (0.031) | (<0.001) | (0.032) | (<0.001) | (0.049) | (<0.001) | (0.059) | (<0.001) | (0.059) | (<0.001) | (0.064) | (0.001) | (0.061) | (0.001) | (0.082) |

**Table S5** (Continued).

| **Variable** | **Self-rated health status** | | | | **Mental health status** | | | | **2-week health status** | | | | **Chronic disease status** | | | |
| --- | --- | --- | --- | --- | --- | --- | --- | --- | --- | --- | --- | --- | --- | --- | --- | --- |
|  | **EI** | | **WI** | | **EI** | | **WI** | | **EI** | | **WI** | | **EI** | | **WI** | |
|  | Pure_  explained | Pure_  Unexplained | Pure_  explained | Pure_  Unexplained | Pure_  explained | Pure_  Unexplained | Pure_  explained | Pure_  Unexplained | Pure_  explained | Pure_  Unexplained | Pure_  explained | Pure_  Unexplained | Pure_  explained | Pure_  Unexplained | Pure_  explained | Pure_  Unexplained |
| **Residence (Reference group: Rural)** | | | | | | | | | | | | | | | | |
| Urban | <0.001 | 0.007 | <0.001 | 0.007 | 0.003** | -0.007 | 0.004** | -0.005 | -0.001 | 0.020 | -0.001 | 0.016 | -0.003* | 0.028 | -0.003** | 0.039 |
|  | (0.001) | (0.021) | (0.001) | (0.022) | (0.001) | (0.036) | (0.002) | (0.044) | (0.001) | (0.036) | (0.001) | (0.039) | (0.001) | (0.033) | (0.002) | (0.045) |
| **Ethnicity (Reference group: Other ethnicities)** | | | | | | | | | | | | | | | | |
| Han Chinese | -0.006*** | -0.124* | -0.006*** | -0.134** | 0.006* | 0.027 | 0.007* | 0.040 | -0.005 | -0.106 | -0.005 | -0.104 | <-0.001 | 0.118 | -0.001 | 0.174 |
|  | (0.002) | (0.064) | (0.002) | (0.068) | (0.003) | (0.105) | (0.004) | (0.124) | (0.003) | (0.094) | (0.003) | (0.100) | (0.003) | (0.096) | (0.003) | (0.128) |
| **Marital status (Reference group: Married/Cohabiting)** | | | | | | | | | | | | | | | | |
| Single | -0.006*** | 0.019 | -0.007*** | 0.021 | -0.007** | 0.102** | -0.010** | 0.125** | -0.005* | 0.077 | -0.006* | 0.084* | -0.001 | 0.043 | -0.002 | 0.052 |
|  | (0.002) | (0.027) | (0.002) | (0.028) | (0.003) | (0.042) | (0.004) | (0.051) | (0.003) | (0.047) | (0.003) | (0.050) | (0.003) | (0.045) | (0.003) | (0.060) |
| Divorced/  Widowed | 0.007** | 0.001 | 0.007** | <0.001 | 0.004 | 0.003 | 0.008 | 0.003 | -0.005 | -0.010 | -0.004 | -0.010 | 0.006 | 0.007 | 0.008 | 0.007 |
|  | (0.003) | (0.003) | (0.003) | (0.003) | (0.005) | (0.006) | (0.005) | (0.008) | (0.004) | (0.008) | (0.005) | (0.009) | (0.004) | (0.005) | (0.005) | (0.007) |
| **Years of education** | 0.006 | 0.033 | 0.006 | 0.036 | 0.019** | -0.095 | 0.025** | -0.111 | 0.023*** | -0.018 | 0.024*** | -0.008 | 0.008 | 0.059 | 0.009 | 0.115 |
|  | (0.005) | (0.054) | (0.006) | (0.057) | (0.009) | (0.087) | (0.010) | (0.103) | (0.009) | (0.111) | (0.009) | (0.119) | (0.008) | (0.114) | (0.010) | (0.153) |
| **Agricultural occupation (Reference group: Non-agricultural)** | | | | | | | | | | | | | | | | |
| Agricultural | 0.005*** | 0.035* | 0.006*** | 0.037* | 0.007*** | 0.055* | 0.008*** | 0.066* | 0.004** | 0.015 | 0.005** | 0.015 | 0.004** | -0.002 | 0.005** | -0.015 |
|  | (0.001) | (0.018) | (0.002) | (0.019) | (0.002) | (0.031) | (0.002) | (0.037) | (0.002) | (0.034) | (0.002) | (0.037) | (0.002) | (0.032) | (0.002) | (0.044) |
| **Self-rated social status (Reference group: Average)** | | | | | | | | | | | | | | | | |
| Relatively low | 0.002 | 0.013 | 0.002 | 0.013 | 0.013*** | -0.013 | 0.014*** | -0.010 | 0.005 | 0.018 | 0.005 | 0.019 | -0.005* | 0.044 | -0.006* | 0.066 |
|  | (0.002) | (0.019) | (0.002) | (0.020) | (0.004) | (0.031) | (0.004) | (0.037) | (0.003) | (0.039) | (0.003) | (0.042) | (0.003) | (0.038) | (0.004) | (0.051) |
| Relatively high | -0.009*** | -0.023* | -0.010*** | -0.024* | -0.009** | -0.002 | -0.010** | -0.002 | -0.004 | -0.003 | -0.004 | -0.002 | 0.003 | 0.010 | 0.003 | 0.017 |
|  | (0.003) | (0.013) | (0.003) | (0.014) | (0.004) | (0.017) | (0.005) | (0.020) | (0.004) | (0.019) | (0.004) | (0.020) | (0.004) | (0.017) | (0.005) | (0.024) |
| **Past-month smoking (Reference group: No)** | | | | | | | | | | | | | | | | |
| Yes | <0.001 | -0.009 | <0.001 | -0.009 | <0.001 | 0.016 | <-0.001 | 0.020 | <0.001 | 0.015 | <0.001 | 0.016 | <0.001 | -0.018 | <0.001 | -0.026 |
|  | (0.001) | (0.015) | (0.001) | (0.016) | (<0.001) | (0.026) | (<0.001) | (0.031) | (<0.001) | (0.024) | (<0.001) | (0.026) | (<0.001) | (0.024) | (<0.001) | (0.033) |
| **Frequent past-month alcohol use (Reference group: No)** | | | | | | | | | | | | | | | | |
| Yes | <-0.001 | -0.001 | <-0.001 | -0.001 | <0.001 | -0.001 | <0.001 | -0.001 | <0.001 | 0.015 | <0.001 | 0.016 | <-0.001 | -0.008 | <-0.001 | -0.015 |
|  | (<0.001) | (0.009) | (<0.001) | (0.009) | (<0.001) | (0.013) | (<0.001) | (0.016) | (<0.001) | (0.013) | (<0.001) | (0.014) | (<0.001) | (0.014) | (<0.001) | (0.019) |
| **Nap habit (Reference group: No)** | | | | | | | | | | | | | | | | |
| Yes | -0.002 | -0.003 | -0.002 | -0.004 | -0.002 | 0.053 | -0.003 | 0.064 | -0.010*** | 0.030 | -0.011*** | 0.034 | -0.010*** | -0.025 | -0.012*** | -0.028 |
|  | (0.002) | (0.022) | (0.002) | (0.023) | (0.003) | (0.036) | (0.004) | (0.043) | (0.004) | (0.045) | (0.004) | (0.049) | (0.003) | (0.046) | (0.004) | (0.061) |

**Table S5** (Continued).

| **Variable** | **Self-rated health status** | | | | **Mental health status** | | | | **2-week health status** | | | | **Chronic disease status** | | | |
| --- | --- | --- | --- | --- | --- | --- | --- | --- | --- | --- | --- | --- | --- | --- | --- | --- |
|  | **EI** | | **WI** | | **EI** | | **WI** | | **EI** | | **WI** | | **EI** | | **WI** | |
|  | Pure_  explained | Pure_  Unexplained | Pure_  explained | Pure_  Unexplained | Pure_  explained | Pure_  Unexplained | Pure_  explained | Pure_  Unexplained | Pure_  explained | Pure_  Unexplained | Pure_  explained | Pure_  Unexplained | Pure_  explained | Pure_  Unexplained | Pure_  explained | Pure_  Unexplained |
| **Weekly exercise (Reference group: No)** | | | | | | | | | | | | | | | | |
| Yes | -0.001 | 0.008 | -0.001 | 0.008 | -0.004*** | -0.009 | -0.004*** | -0.011 | -0.002 | -0.010 | -0.002 | -0.012 | -0.001 | 0.005 | -0.002 | 0.001 |
|  | (0.001) | (0.014) | (0.001) | (0.015) | (0.001) | (0.024) | (0.002) | (0.029) | (0.001) | (0.031) | (0.001) | (0.034) | (0.001) | (0.031) | (0.001) | (0.042) |
| **Life satisfaction (Reference group: Somewhat satisfied)** | | | | | | | | | | | | | | | | |
| Dissatisfied/  Neutral | 0.004 | -0.010 | 0.005* | -0.011 | 0.006 | -0.010 | 0.003 | -0.003 | 0.002 | -0.005 | 0.002 | -0.009 | 0.003 | -0.074 | 0.004 | -0.105 |
|  | (0.003) | (0.024) | (0.003) | (0.026) | (0.005) | (0.041) | (0.005) | (0.048) | (0.004) | (0.049) | (0.004) | (0.052) | (0.004) | (0.048) | (0.005) | (0.064) |
| Very satisfied | <0.001 | -0.001 | 0.001 | <-0.001 | -0.001 | 0.011 | -0.003 | 0.014 | 0.008** | 0.009 | 0.008** | 0.010 | 0.007** | -0.036 | 0.009** | -0.054 |
|  | (0.002) | (0.017) | (0.002) | (0.018) | (0.003) | (0.023) | (0.004) | (0.028) | (0.004) | (0.032) | (0.004) | (0.035) | (0.003) | (0.032) | (0.004) | (0.043) |
| **Cooking water source (Reference group: Non-tap water)** | | | | | | | | | | | | | | | | |
| Tap water | <-0.001 | 0.015 | <-0.001 | 0.019 | -0.003** | 0.081 | -0.004** | 0.095 | -0.001 | 0.036 | -0.001 | 0.042 | <-0.001 | 0.027 | <-0.001 | 0.018 |
|  | (<0.001) | (0.032) | (<0.001) | (0.033) | (0.001) | (0.051) | (0.002) | (0.062) | (0.001) | (0.054) | (0.001) | (0.058) | (0.001) | (0.048) | (0.001) | (0.067) |
| **Cooking fuel type (Reference group: Non-clean fuels)** | | | | | | | | | | | | | | | | |
| Clean fuels | 0.001 | -0.023 | 0.001 | -0.022 | -0.001 | -0.027 | <-0.001 | -0.039 | -0.002 | 0.017 | -0.002 | 0.020 | <-0.001 | -0.072 | <0.001 | -0.111* |
|  | (0.001) | (0.031) | (0.001) | (0.033) | (0.002) | (0.052) | (0.002) | (0.062) | (0.002) | (0.052) | (0.002) | (0.055) | (0.002) | (0.046) | (0.002) | (0.063) |
| **Household size** | -0.013*** | 0.022 | -0.015*** | 0.029 | -0.014** | 0.072 | -0.013* | 0.081 | -0.013** | 0.006 | -0.013* | 0.001 | -0.001 | 0.077 | <-0.001 | 0.087 |
|  | (0.004) | (0.046) | (0.005) | (0.048) | (0.006) | (0.062) | (0.007) | (0.073) | (0.007) | (0.071) | (0.007) | (0.076) | (0.006) | (0.052) | (0.007) | (0.073) |
| **Basic social medical insurance enrollment (Reference group: Not enrolled)** | | | | | | | | | | | | | | | | |
| Enrolled | -0.001 | -0.096 | -0.001 | -0.103 | <0.001 | -0.081 | <0.001 | -0.104 | -0.001 | -0.028 | -0.001 | -0.036 | -0.002* | -0.064 | -0.002* | -0.069 |
|  | (0.001) | (0.069) | (0.001) | (0.073) | (0.001) | (0.127) | (0.001) | (0.153) | (0.001) | (0.125) | (0.001) | (0.134) | (0.001) | (0.104) | (0.001) | (0.140) |
| **Level of medical expertise of the visited institution (Reference group: Fair)** | | | | | | | | | | | | | | | | |
| Low | <0.001 | 0.004 | <0.001 | 0.004 | 0.003* | -0.006 | 0.003* | -0.008 | 0.001 | -0.020 | 0.001 | -0.022 | 0.004** | -0.016 | 0.004** | -0.016 |
|  | (0.001) | (0.011) | (0.001) | (0.012) | (0.002) | (0.016) | (0.002) | (0.019) | (0.001) | (0.016) | (0.001) | (0.018) | (0.002) | (0.015) | (0.002) | (0.020) |
| High | -0.005*** | 0.003 | -0.005*** | 0.003 | -0.005* | -0.018 | -0.006* | -0.026 | -0.006** | -0.010 | -0.007** | -0.009 | -0.001 | 0.029 | -0.002 | 0.047 |
|  | (0.002) | (0.022) | (0.002) | (0.023) | (0.003) | (0.034) | (0.003) | (0.042) | (0.003) | (0.043) | (0.003) | (0.046) | (0.003) | (0.043) | (0.003) | (0.058) |
| Year FE | Yes | Yes | Yes | Yes | Yes | Yes | Yes | Yes | Yes | Yes | Yes | Yes | Yes | Yes | Yes | Yes |

*, **, and *** denote significance at the 10%, 5%, and 1% levels, respectively. *PI* Personal Income. *PCHI* Per Capita Household Income. *MIRR* Medical Insurance Reimbursement Ratio.
